# Aptamer Sequence Optimization and Its Application in Food Safety Analysis

**DOI:** 10.3390/foods14152622

**Published:** 2025-07-26

**Authors:** Xinna Qin, Lina Zhao, Yang Zhang, Jiyong Shi, Haroon Elrasheid Tahir, Xuechao Xu, Kaiyi Zheng, Xiaobo Zou

**Affiliations:** 1 School of Food and Biological Engineering, Jiangsu University, Zhenjiang 212013, China; 2 School of Food Science and Engineering, Yangzhou University, Yangzhou 225127, China

**Keywords:** aptamer, sequence, optimization, target, detection

## Abstract

Aptamers are single-stranded DNA or RNA oligonucleotides screened by systematic evolution of ligands by exponential enrichment (SELEX) methods, which are widely used in food analysis. Aptamers have the advantages of low molecular weight, ease of preparation, simplicity of chemical modification, and structural stability. Aptamers generated by SELEX are typically 80–100 bases in length, and the affinity of the aptamer can be improved by sequence optimization. Methods of aptamer optimization commonly include truncation, mutation, and chemical modification, and molecular docking, molecular dynamics, circular dichroism, and isothermal titration to assess often the binding performance of the aptamer to the target. Optimized aptamers usually enhance the affinity of the aptamer for the target and increase its sensitivity in the detection of pesticides, heavy metals, fungal toxins, pathogenic bacteria, and other objects. This paper focuses on truncation, mutation, chemical modification, the introduction of rare nucleotides, and computer-aided design. It provides an overview of non-immobilized optimization metrics.

## 1. Introduction

Aptamers have been evolving phylogenetically for more than 20 years. In 1990, Larry Gold and Andrew Ellington respectively discovered them, using systematic evolution of ligands by exponential enrichment (SELEX) methods [1,2]. To date, a variety of DNA or RNA aptamers have been identified for various targets, such as small molecules [3,4,5], large molecules [6], viruses [7], cells [8,9], and ions [10,11], with high affinity and specificity [12,13], and have been applied to a variety of assays, such as in the fields of chemical analysis and bioanalysis [14,15]. Furthermore, aptamers have been utilized to develop portable detection devices, providing new tools for immediate detection.

Aptamers are being used with increasing frequency in food analysis. Contaminated food is often found to contain hazardous substances, including mycotoxins [16], pesticides [17], and bisphenols [18]. In response to the demand for rapid, sensitive, and efficient detection, nucleic acid aptamers biosensors have been widely adopted due to their significant advantages [19]. The most prevalent of these is the detection of mycotoxins in food, as exemplified by the most toxic aflatoxin (AFB1), which has garnered significant interest among a broad spectrum of researchers. Li et al. [20] obtained the truncated aptamer AF11-2 after several rounds of improved pro-chromatographic SELEX strategy analysis, which significantly enhanced the fluorescence ability of AFB1. The aptamer was able to sensitively detect AFB1 with a detection limit as low as 42 nM. Fungal poisoning constitutes the predominant category of food poisoning incidents. Gao et al. [21] developed a magnetic bead enzyme immunoassay using α-gooseberry mycoplasma in Gooseberry. Utilizing the original aptamer as the recognition element, the detection limit was 369 μg/L. Currently, some merchants illegally added some drugs to enhance the function of beverages. Wang et al. [22] developed a fluorescence method combined with a label-free aptamer to detect metronidazole and ibuprofen in soft drinks.

However, the aptamer sequences screened by the traditional SELEX library are long, which cannot meet the requirements of high sensitivity and high affinity in practical work. In order to enhance the sensitivity and affinity and reduce expense, it is necessary to optimize the sequence of the aptamer [23]. The optimized aptamer exhibits higher stability and a large binding affinity to the target. In the current work, common optimization strategies include truncation [24], splitting [25], mutation [26], bivalent or multivalent construction [27], and aptamer chemical modification [28]. The optimization methods referred to in this paper are all applied after SELEX procedure. The paper is presented with a focus on truncation, mutation, chemical modification, introduction of rare nucleotides and computer-aided design. The truncation strategy has been identified as the preferred approach for aptamer optimization [29].

In order to ascertain whether the modified aptamer sequence is capable of maintaining the high affinity and specific binding to the target, the aptamer sequence is required to be evaluated by some optimization metrics [30]. The classification of optimization metrics is based on whether the aptamer requires immobilization, which is denoted as either immobilized or non-immobilized strategies. Immobilization strategies, including surface plasmon resonance [31,32], enzyme-linked aptamer resonance [33], and biosphere interferometry [34], frequently encounter limitations in sensitivity, substantial equipment and maintenance costs, and intricate operational procedures. Non-immobilization strategies encompass a range of techniques, including circular dichroism [35], isothermal titration [36], microcalorimetric thermophoresis [37], and fluorescence analysis [38,39]. The results of the optimization metrics can guide subsequent aptamer research. And this paper focuses on an over-view of non-immobilized optimization metrics.

This review systematically summarizes recent developments in sequence optimization strategies and key optimization parameters for nucleic acid aptamers. It also highlights the practical applications of these optimized aptamers, aiming to provide a comprehensive reference that supports the future development of nucleic acid aptamer-based biosensors and their applications in food safety and quality detection.

## 2. Aptamer Sequence Optimization Strategies

**Figure 1:**
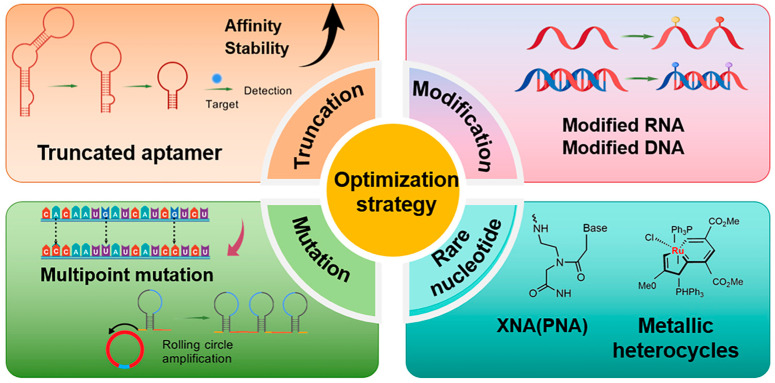
Overview of aptamer sequence optimization strategies.

Aptamer optimization entails the meticulous refinement of the aptamer sequence to enhance its affinity and specificity for a specific target. Screening techniques, such as SELEX, can be used to screen aptamers from random nucleic acid libraries with high affinity for the target molecule. However, the addition of aptamer optimization on top of screening can shorten the optimization time and further improve the affinity between the aptamer and the target. The optimization strategies employed in contemporary research encompass truncation, mutation, and chemical modification, along with the incorporation of classical nucleotides for modification. The advent of artificial intelligence has also elevated computer-aided design to a prominent position. A selection of the optimization strategies that have been documented in recent years is presented in Figure 1. The truncation strategy has also seen increased utilization among researchers, demonstrating notable efficacy.

### 2.1. The Strategy of Truncation

The aptamer truncation strategy, defined as the identification of the key binding sites of the aptamer [40], involves the removal of non-essential components from the aptamer. This process results in the identification of a more effective core region of the aptamer, which can include structures such as stem loops and bumps. Usually, the cost of synthesizing an aptamer increases with the increase in the number of bases [19]. Meanwhile, the long chain bases can weaken target-aptamer recognition. Aptamer recognition relies on specific sequence conformations, but redundant long-chain aptamer sequences form complex structures that may block target binding sites and reduce binding capacity. Therefore, suitable truncation of the aptamer can realize the potential of the long-chain aptamer.

The efficacy of truncated aptamers is demonstrated by their enhanced affinity for the target and increased stability [41]. In an in vitro screening and optimization of high-affinity aptamers for the milk allergen α-lactalbumin, the *K*_d_ of the truncated aptamer was 14.05 ± 4.15 nM, while the *K*_d_ of the full length sequence was 92.6 ± 18.0 nM, indicating a six-fold increase in the binding affinity of the truncated aptamer [42]. Deoxynivalenol, or DON, is a mycotoxin produced by the fungus Fusarium oxysporum, which can infect wheat, corn, barley, and other crops. Han et al. [43] designed an aptamer capable of detecting deoxynivalenol. This aptamer was modified by shortening its bases from 80 to 40 and decreasing its *K*_d_ from nearly 80 nmol/L to 30.38 nmol/L. These modifications resulted in a significant enhancement of the aptamer’s binding affinity to its target. Nertilmicin (NET) is a veterinary antibiotic importance and has a significant-prevalence in the fields of aquaculture and animal husbandry. In a seminal study, Jing et al. [44] initially truncated the NET aptamer of 80 nucleotides, which had been screened, to one of 60 nucleotides (N60). They then proceeded to explore the binding site and further truncated it to 20 nucleotides (N20). Ultimately, they selected N20 for the highest affinity. The binding sites between aptamers and targets include the formation of hydrogen bonds between bases and target functional groups, secondary and tertiary structure sites, and hydrophobic and electrostatic interaction sites. Utilizing the molecular docking strategy [45], the inter-molecular interaction forces and interaction sites between the target and aptamer were analyzed, and the long-chain aptamer was gradually optimized to enhance the binding affinity. In the study by Nguyen et al. [46], aptamer sensors for truncation and molecular docking with glucose, cocaine, and theophylline were analyzed. The removal of nucleotides from the 3′ and 5′ ends of the aptamers resulted in a better response from the truncated aptamers compared to the original sequence.

### 2.2. The Strategy of Mutation

The aptamer mutagenesis strategy involves the modification of the aptamer se-quence to enhance its affinity and stability for a specific target, thereby increasing its bioavailability. The recognition site of the aptamer is often specified through targeted mutagenesis [47], which involves the introduction of base mutations at specific positions of the aptamer. This approach is employed to explore new binding sites or to enhance the affinity of existing binding sites. Gao et al. [48] employed the aptamer OBA3, a dual-target ochratoxin A (OTA) and norfloxacin (NOR) aptamer, to illustrate the study and exploration of the binding specificity mechanism. Through subsequent experiments, it was evidenced that substituting the T-base at position 15 with a C-base could enhance the recognition specificity of the aptamer for NOR while eradicating its binding affinity for OTA.

The binding ability can be enhanced through the construction of bivalent [49] or multivalent aptamers [50]. Zhou et al. [51] demonstrated the distinction between in-SELEX and post-SELEX techniques in the context of aptamer modification. The in-SELEX strategy involves the direct introduction of modified nucleotides as substrates for the synthesis of aptamers in the SELEX process. This method is capable of screening out the aptamers that have already undergone modification, thereby enhancing their performance and reducing the time and financial resources in post-processing. Post-SELEX modifications are introduced at various positions of the aptamer (e.g., bases, sugar loops, phosphate groups, etc.) during solid-phase synthesis. While both approaches have been demonstrated to enhance nuclease resistance and affinity to a certain extent, they remain subject to continuous adjustment and optimization. Sun et al. [52] discovered that the 6-base loop structure composed of CAGTGA may be the key region for target binding after truncation and optimization of *Vibrio parahaemolyticus* nucleic acid aptamer. The authors then used fixed-point mutagenesis to optimize this part of the sequence. They found that when other bases replaced this part of the sequence, the aptamer sequence’s affinity was reduced. This result proved that this structure is important for the process of hazard identification. The utility of these mutations lies in their potential to facilitate scientific research by allowing for the discernment of the binding strength of the original aptamer sequence. This insight, in turn, paves the way for a more in-depth exploration of the underlying binding mechanism. In the event that the binding ability is augmented following the mutation, it can be utilized directly in the design of new aptamer sequences.

Currently, the optimization of aptamers using mutation strategies has produced important positive effects in medical treatment, including substantial advancements in the development of nucleic acid aptamer-targeted antitumor drugs, and the idea of how to take advantage of this progress to promote the research of mutant aptamers in other bioanalytical fields is also a current issue that needs to be focused. In the field of food science, aptamers have the potential to be useful in detecting mutated foods, such as genetically modified foods and diseased animal tissues, as well as determining the authenticity of plants.

### 2.3. Non-Base Chemical Modification Strategy

Aptamer chemical modification is the modification of nucleic acid aptamers by chemical means [53]. There are two main sections: base (adenine (A), guanine (G), cytosine (C), thymine (T), uracil (U)) chemical modifications, which are placed in Section 2.4 for detailed introduction; and non-base (ribose, phosphate) chemical modifications. Non-basic modifications encompass end modifications, such as 3′- and 5′-end modifications [54], the addition of a protecting group, or the attachment of other components, which contribute to enhancing the stability of the aptamer. Glycocyclic modifications [55] have been used to improve the resistance of aptamers to nuclease degradation. The modification of the phosphate backbone, which involves the introduction of fluorine, amino, and methyl groups to the ribose or deoxyribose of the aptamer, or the alteration of the phosphodiester linkage to a thiodiphosphate bond, has been shown to enhance the aptamer’s resistance to degradation and contribute to its stability [56].

Presently, alternative strategies for other substances have been proposed based on sugars in nucleotides. Accordingly, a variety of heterogeneous nucleotides (XNA) have been postulated, including glycol nucleic acid (GNA), cyclohexene nucleic acid (CeNA), hexanol nucleic acid (HNA), click nucleic acid (CAN), threonine nucleic acid (TNA), arabinose nucleic acid (ANA), and fluoroarabinose nucleic acid (F-ANA), among others [57]. The primary structural characteristics of these nucleotides are delineated in Table 1. A primary benefit of XNA is its improved biostability and remarkable target affinity, in addition to its observed catalytic activity in certain instances. A significant advantage of XNA over DNA and RNA is its higher stability to nucleases, which gives XNA the potential for a wide range of applications in several areas of molecular biology [58]. It has been demonstrated that FANA aptamers exhibit an affinity for the receptor-binding domain (RBD) and SARS-CoV-2 S proteins that is comparable to that of previous DNA aptamers. In addition, XNA aptamers have been shown to bind their targets with very high affinity and are fully RNase-resistant [59].

These XNAs are widely used in medicine to detect and treat diseases, such as cancer cells, bacteria (mainly tuberculosis), and virus (AIDS) drug resistance. In the food sector, the misuse of antibiotics and antimicrobials in food cultivation has led to the mutation of bacteria and fungi, which in turn has led to a sustained increase in the emergence of multi-drug resistant pathogens and new infectious diseases [60]. Research has proven that DNA modifications, histone modifications, rRNA methylation, and non-coding RNA expression regulation influence the development and enrichment of antimicrobial resistance (AMR). These modifications modulate various biological processes, including gene expression, bacterial phase transitions, tolerance, and persistence. These mechanisms function in concert to meticulously regulate bacterial adaptation and resistance to antibiotics [61]. Utilizing these XNAs to detect antimicrobials within food products facilitates the timely identification of drug-resistant bacteria, thereby ensuring the quality and safety of food products [62].

In addition to bacteria and fungi in agriculture, mutations of viruses are common in farming, and these mutated viruses are very susceptible to zoonotic diseases. European branch 2.3.4.4b Influenza A (H5N1) viruses can be replicated in bovine cells and heat-inactivated in semi-skimmed or whole milk and produce dependent mutations. Influenza A (H5N1)-associated conjunctivitis and upper respiratory flulike illness have been reported in dairy farm workers [63]. In raw vegetables, noroviruses and other viruses have been observed to cause human food poisoning through cross-infection and other means of transmission [64]. The potato virus genome contains highly variable regions that exhibit a preference for the accumulation of nucleotide substitutions. This attribute renders a broad spectrum of plants from diverse genera and plant families across the globe susceptible to potato viruses, which in turn results in significant economic losses [65]. If these XNAs can be used for the early identification of viral mutations in the farming industry, and based on their viral mutation patterns, the pathogenicity modes, detection schemes and targeted treatment can be predicted. Meanwhile, corresponding countermeasures can be taken, and the risk of zoonotic diseases will be significantly reduced.

Furthermore, there has been an increase in the consumption of genetically modified (GM) foods, yet consumers have expressed concerns regarding the safety of these products. Using XNAs to identify GM foods and predict their toxicity can protect consumer health and ensure informed choices about GM ingredients.

### 2.4. Introduction of Rare Nucleotides

Rare nucleotides are nucleotides that are formed by substituting rare bases for the original bases, and these rare bases are mainly: modifications of natural bases, and introduction of non-natural bases.

**Figure 2:**
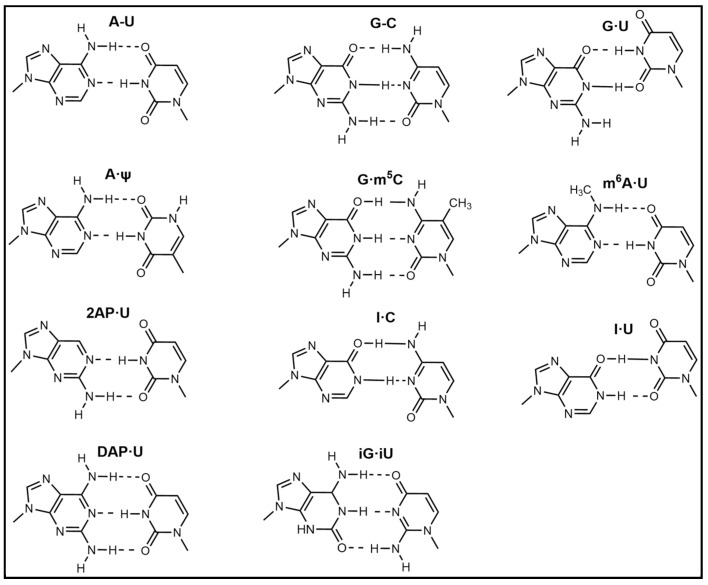
Base pairing diagram [67]. Reproduced from [Melissa C Hopfinger, Charles C Kirkpatrick and Brent M Znosko, Nucleic Acids Research, 2020], with permission from [Oxford University Press].

Modification of natural bases entails chemical structural alterations in the conventional bases (adenine (A), guanine (G), cytosine (C), thymine (T), and uracil (U)) without compromising the structure of the heterocyclic parent ring [66]. These changes can alter hydrogen bonding or hydrophobic interactions and improve the binding affinity of the aptamer. Commonly modified bases include pseudouridine (Ψ), hypoxanthine (Ι), 5-methylcytosine (m^5^C), N6-methyladenosine (m^6^A), and 5-hydroxymethylcytosine (hm^5^C) [67]. These bases bear a resemblance to conventional bases and are capable of undergoing base complementary pairing. Figure 2 presents a schematic representation of prevalent modified base pairings.

The modification of natural bases has been demonstrated to expand the variety of nucleotides and facilitate the screening of superior aptamers in the context of food testing. In the context of base modification, m^6^A represents one of the most prevalent modifications. Shi et al. [68] assayed demethylase activity and designed a demethylation variable aptamer probe using a computer-assisted approach. This approach was developed for the efficient screening of potential m^6^A (N6-methyladenosine) modification sites and the modification of m^6^A on the aptamer. The findings of this study may offer novel concepts and establish a shared framework for the advancement of dynamic imaging analysis of additional epigenetic key enzymes in vivo. In the chemical modification of nucleotides, the volume size has been demonstrated to alter the binding orientation of the aptamer to the target site. Furthermore, the flexible modification of position and quantity enhances the performance of the aptamer. Murry et al. [69] employed the case of thrombin binding aptamer to elucidate the impact of size, number, and position of modifying groups on aptamer binding ability. Concurrently, they combined data mining techniques to explore the optimal modification method.

Modifications of the natural bases can also enhance the optical-electrical activity of the heterocycle, thereby improving the sensitivity of the assay. Saito and Hudson et al. [70] provided a comprehensive overview of the design, synthesis, and application of purine nucleoside analogues with fluorescently modified nucleobases. These include C7, C8, C2, C3, and N6 modified fluorescent purine nucleosides. It has been established that moderate or significant structural modifications of nucleobases may produce the production of fluorescence. Consequently, the fluorescence intensity of the aptamer can be augmented by heterocyclic modification, which facilitates the utilization of nucleic acid sequences for the detection of potentially hazardous substances in food. In addition to fluorescent groups, Raman-active groups (e.g., triple bonds with a characteristic response in the Raman silent region of 2000–2500 cm^−1^ as well as cumulative double bonds [71]), and electrochemical groups [72] introduce aptamers by combining rare bases, which can also increase the sensitivity of the corresponding detection methods.

It has also been found that modified natural bases used in aptamers enhance the binding of the aptamer to the target, target specificity and exclusivity, and stability of the aptamer. Furthermore, it has been observed that aptamers exhibit distinct *K*_d_ values in buffer compared to serum [73]. Complex biological substrates, such as milk, egg whites, and fruit juices, are also present in these foods. The attachment of aptamers with modified natural bases has been demonstrated to facilitate the monitoring of harmful substances in complex mechanisms. Meanwhile, aptamers with a modified natural base can be applied to the analysis of proteins, such as salivary amylase [74]. According to the findings of the aforementioned research, aptamers containing modified natural bases can be designed to detect toxic proteins in food, such as anti-nutritional proteins, allergenic proteins, and microbial toxin proteins. Additionally, the aptamer with modified natural bases has the capacity to generate a robust interaction with viruses, including those that cause diseases such as SARS-CoV-2 [75]. Consequently, the aptamer with modified natural bases can also be utilized to expedite the detection of various viruses in raw food materials, including foot-and-mouth disease virus, avian influenza virus, and hepatitis A virus. Furthermore, in the context of DNA aptamers with modified natural base pairs, their potential applications include the use as hybridization probes in DNA diagnostic tools and as imaging and detection tools for target DNA molecules [76]. In the future, molecular imaging in the field of food research will explore the sterilization mechanism, the effect of bacterial inhibition, and whether resistance occurs when biocides are added. It will also facilitate the early identification of drug-resistant bacteria.

**Figure 3:**
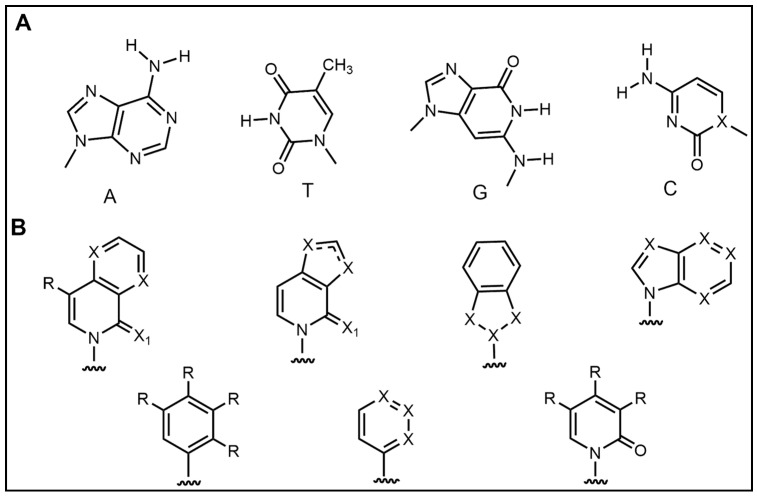
Natural versus unnatural bases [78]; (**A**) natural bases; (**B**) unnatural nucleotide substitutions. X: = heteroatom substitution; R: = functional group. Reproduced from [Aaron M. Leconte, Gil Tae Hwang, Shigeo Matsuda, Petr Capek, Yoshiyuki Hari and Floyd E. Romesberg, Journal of the American Chemical Society, 2008], with permission from [American Chemical Society].

In addition to the modification of natural bases, the parent ring can be constructed from other heterocycles of non-natural bases. These heterocycles can also be substituted for natural bases to construct aptamers, as demonstrated in Figure 3. Chen et al. [77] introduced the artificial nucleotide (AN) for the construction of AN-Apt-FET aptamers for exosomes, which improved the sensitivity and stability in complex biological environments.

D5SICS:dMMO2 is a promising non-natural base pair that exhibits synthesis and extension efficiencies analogous to those of natural base pairs [78]. This base pair exhibits superior performance in terms of fidelity and efficiency when compared to other reported non-natural base pairs, thereby facilitating significant advancements in the expansion of the genetic code. Furthermore, the non-natural base pair technology is anticipated to contribute to advancements in DNA storage, biomolecular labeling, and novel drug development, thereby facilitating further breakthroughs in the domain of synthetic biology and biotechnology [79]. The structural characteristics of different unnatural bases are enumerated in Table 2.

There are already applications of non-natural base pairs in medical diagnostics [80], including aptamers with natural bases and alternative heterocycles for detecting cancer cells and exploring their proliferative capacity [81]. These findings can be extrapolated to the realm of food safety, particularly with regard to the detection of bacteria and the subsequent monitoring of their potential proliferative capacity and metabolic activity. Based on the research, the capacity to promptly identify potentially deleterious bacteria in foods can be achieved. The employment of aptamer with non-natural bases can also facilitates the observation of nucleic acid life processes within the cell, including the regulation of transcription, translation, transport, and other relevant processes by nucleic acids [82]. Subsequently, in the domain of food analysis, the screening of bacterial pathogenicity and putrefaction ability is conducted. The early identification of abnormal harmful bacteria after mutation is of great significance in the field of ensuring food safety and human health.

**Figure 4:**
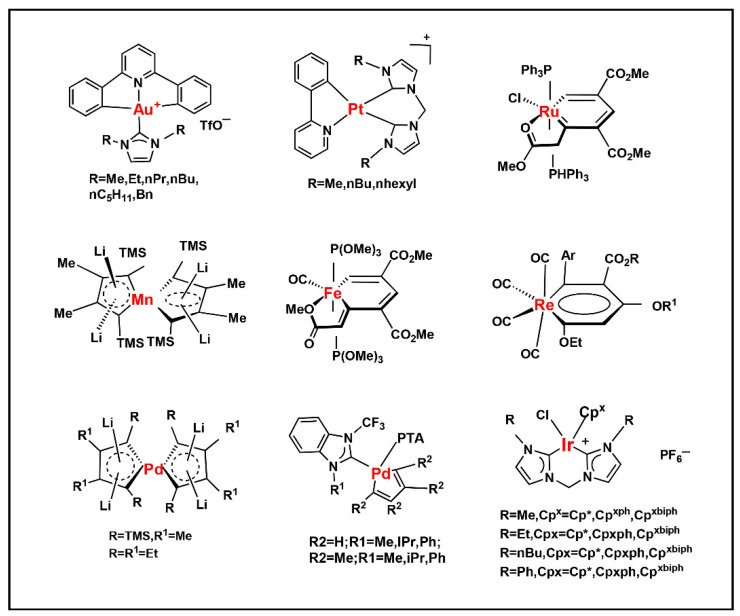
Structural formulae of some metal heterocycles [86,87].

In recent years, the discovery of metal-organic heterocyclic compounds has garnered significant attention due to their distinctive properties [83]. It has been demonstrated that certain metal-organic compounds possess enhanced antitumor properties [84]. Substituting natural bases with such metal loadings could facilitate not only detection but also potential antitumor functionality. Among them, cisplatin is widely used in the clinical treatment of cancer, and some other metal compounds replace cisplatin by changing the nature of the side groups. Harlepp et al. [85] synthesized and characterized platinum (II) complexes stabilized by pyridine and N-heterocyclic carbene (NHC) ligands. The researchers found that the compounds exhibited high in vitro antiproliferative activity against cancer cell lines and that the platinum complexes were able to be embedded in base pairs. This allowed the DNA to bridge with platinum complexes to form a highly compressed, dense structure and improve the stability of DNA. In addition to platinum, other metals, including ruthenium, rhodium, and palladium, have been observed to interact with DNA through a variety of mechanisms, such as cross-linking, insertion, and reactive oxygen species generation [86,87]. Figure 4. presents a selection of metal heterocycles.

### 2.5. Computer-Assisted Design of Aptamer

The advent of big data technology has led to a marked increase in the application of bioinformatics data mining methods to the optimization of aptamers. A variety of machine learning-based aptamer design models have emerged, including the Potts model, RaptGen model, and AptaDiff model. Aptadiff has been demonstrated to leverage motif-dependent potential embeddings from variant autoencoders to generate aptamers that exceed the limitations of high-throughput sequencing data. Furthermore, the optimized aptamers exhibit superior binding affinity compared to those via SELEX [88].

The integration of artificial intelligence and machine learning algorithms to predict and optimize aptamer sequences is emerging as a prominent research direction [89]. This method primarily involves the modeling of a substantial volume of existing aptamer data and their affinities, with the subsequent identification of aptamer sequences with enhanced binding capabilities. In a related study, researchers used particle display (PD) to classify a library of aptamers based on affinity. They then used the data to train a machine-learning model to predict affinity. This helped to shorten 70% of truncated aptamers in design [90]. Mousivand et al. [91] successfully developed a new variant, F20-T, with the help of a parental aptamer design combined with computational simulation using a genetic algorithm for truncation. Test strips based on F20 exhibit high sensitivity in comparison to the truncated aptamers. The computer-assisted strategy enables the selection of broad-spectrum aptamers that target organophosphorus pesticides. This demonstrates its superiority for monitoring pesticide residues in food and the environment [92]. These results allow for the development of various aptamer sensing platforms sustainably and cost-effective.

In addition to aptamer optimization, machine learning platforms have also been utilized by researchers for de novo design of RNA aptamers [93], which can design short RNA aptamers based on fewer known aptamer sequences using the catRAPID algorithm [94]. In a related study, Moussa et al. [95] found that an unsupervised machine learning model called the Potts model can be used to discover new and useful sequences with controlled sequence diversity. Driscoll et al. [96] employed a computer simulation approach to design a novel epithelial cell adhesion molecule (EpCAM) targeting DNA aptamers. These aptamers exhibit high binding and affinity, as well as specificity for EpCAM expressed on cholangiocarcinoma (CCA) cells. These machine learning-based aptamer design methods are applicable to a limited number of targets. Yang et al. [97] combined computer aptamer design with experimental validation to rationally design the aptamer structure to optimize binding affinity, which is conducive to accelerating the development of aptamer-based RNA therapeutics. In addition to affinity, the specificity of aptamer can also be improved by data mining [98,99], which can be used to selective detection of target compounds. The high specificity of aptamer can be achieved by high affinity to target molecule, but low affinity to interference molecules.

Moreover, for the aptamer sequences that are difficult to obtain by SELEX techniques, the machine learning methods can partially replace SELEX techniques through mathematical design to reduce workload. And, the data mining techniques, including molecular docking and molecular dynamics, can be employed to ascertain the binding affinity of aptamers, thereby providing further guidance for experimental design [100]. The particulars of molecular docking and molecular dynamics will be addressed in Section 3.2.1 and Section 3.2.2.

## 3. Evaluation Criteria for Aptamer Optimization

**Figure 5:**
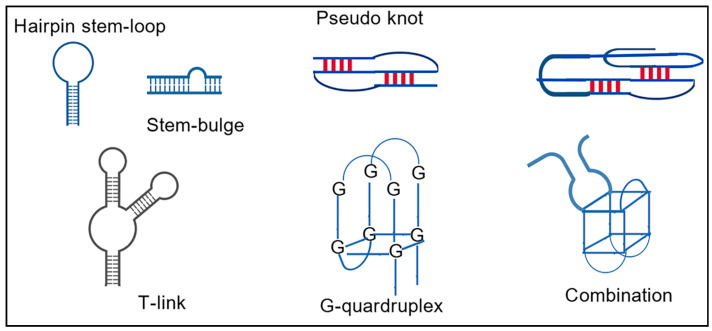
Schematic representation of multiple secondary structures of nucleic acid aptamers [105]. (Hairpin stem-loop: A type of nucleic acid that has a single-stranded stem and a single-stranded loop region. Stem-bulge: A double-stranded stem with an unpaired bulge on one side of the strand. Pseudo knot: It consists of paired bases with the outer stem sequence. T-link: A forked structure (e.g., Y-shape) formed by the intersection of three double-stranded stems. G-quadruplex: A four-stranded planar plane formed by guanine (G)-rich sequences through Hoogsteen hydrogen bonding, creating a nested double-stranded structure. Classified as a secondary structural element despite its three-dimensional nature. Combination: A combination of the above elements (e.g., hairpin + pseudo knot)). Reproduced from [106], with permission from [Elsevier].

The determination of the binding capacity between the aptamer and the target can be achieved through the implementation of experimental methodologies, including circular dichroism, isothermal titration, microcalorimetric thermophoresis, and fluorescence analysis. The utilization of these techniques does not necessitate the solid-phase immobilization of the aptamer or target [101]. Moreover, the experimental process remains uncomplicated. In addition to experimental methods, the advent of computer technology has enabled the study of aptamer-target interactions, such as molecular docking and molecular dynamics, through molecular simulation techniques. These molecular simulation methods can not only reveal the magnitude of the role of the aptamer and the target, but also predict their binding sites, laying the foundation for the subsequent optimization of the aptamer [102]. Among these elements, the secondary structures of aptamers, including pseudoknots, play a key role in RNA aptamers and determine the biological functions of RNA to a certain extent [103]. Aptamers with pseudoknot structures can be used to detect food hazards such as neurotoxins to ensure food safety [104]. As illustrated in Figure 5, the schematic diagrams depict the secondary structures of nucleic acid aptamers.

### 3.1. Experiment Methods

#### 3.1.1. Circular Dichroism

Circular dichroism (CD) is a spectroscopic technique frequently utilized to examine the secondary structure of proteins and other biological macromolecules [107]. The fundamental principle of CD is that the secondary structure of proteins and nucleic acids engenders particular optical properties, leading to the distinct absorption of left- and right-polarized light. By measuring this difference in absorption, information regarding the optical activity of the sample can be obtained, providing insights into its secondary structure. Secondary structures of aptamers, such as stem-loop and hair-pin structures, are imperative for target identification. CD mapping has been shown to yield significant insights into molecular conformations and alterations [108,109], thereby facilitating the evaluation of the binding capacity of aptamers to their respective targets.

Troisi et al. [110] investigated the folding and molecular properties of M08s-1 in solution using a combination of CD, NMR spectroscopy, and natural polyacrylamide gel electrophoresis (PAGE). The effect of variable molecules on the secondary structure was demonstrated by aptamer CD spectroscopy. Liu et al. [42] employed CD spectroscopy to demonstrate that the secondary structure of the truncated aptamer LA-1t contains a hairpin structure. This finding was obtained through in vitro screening and optimization of high-affinity aptamers for the milk allergen α-lactalbumin. Wang et al. [111] designed functionalized QNC (dichloroacid) aptamers and a generic aptamer signal amplification strategy by CD spectroscopy. The results of CD spectroscopy demonstrated that the hybrid-type conformation of the aptamer could be stabilized or induced by binding to QNC.

Recently, Synchrotron Radiation Circular Dichroism (SRCD) can be utilized to examine the interaction of aptamers and target molecules in samples with nanomolar concentrations (µg/mL level) and minimal sample volumes (µL level) [112]. The device is particularly well-suited for the monitoring of metabolite and aptamer interactions in biological samples. Time-Resolved X-ray Circular Dichroism (TRXCD) was employed to explore the dynamics of aptamer-target interactions [113]. This method is particularly well-suited for the study of the early effects of target molecules on aptamer conformation. High Throughput Circular Dichroism (HTCD) is employed for the purpose of forward target finding, wherein aptamers are immobilized to identify targets with which they exhibit a high degree of interaction [114]. The method can also be used for reverse target finding, i.e., aptamer optimization, target fixation, and rapid search for optimal aptamer sequences. CD imaging and microscopy can be used in tissues to identify the role of different nuclear aptamers. In the future, the development of higher-resolution devices has the potential to facilitate the monitoring of organelles and aptamers within cells.

**Figure 6:**
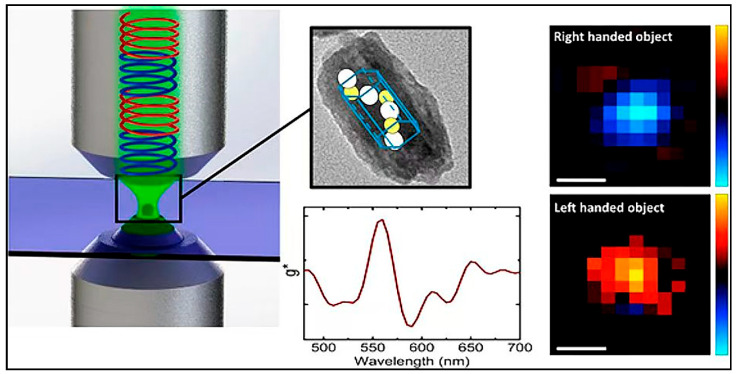
Circular dichroism (CD) technique [116]. Reproduced from [Eitam Vinegrad, Daniel Vestler, Assaf Ben-Moshe, A. Ronny Barnea, Gil Markovich, and Ori Cheshnovsky, Acs Photonics, 2018], with permission from [American Chemical Society].

In most cases, researchers prefer to use techniques to detect small molecular conformational changes and thus assess the low affinity of nucleic acid aptamers [115]. However, the CD technique is primarily applicable to the conformational analysis of biomolecules, such as single proteins or nucleic acids. There may be difficulties in accurately resolving the conformations of nucleic acid aptamers and target molecules in complex systems. Presently, instruments based on CD spectroscopy for assessing the affinity of nucleic acid aptamers are undergoing a shift toward in-creased portability, enhanced intelligence, and accelerated operation. In the future, the reduction of cost, the augmentation of intelligence, and the further acceleration of these instruments will be imperative. The CD technique is illustrated in Figure 6.

#### 3.1.2. Isothermal Titration Calorimetry

Isothermal titration calorimetry (ITC) provides thermodynamic and kinetic information in situ, online, and non-destructively by continuously and accurately monitoring and recording the calorimetric profile of a changing process with a highly sensitive and automated microcalorimeter [117]. ITC can be used to study various methods including, but not limited to: (1) enzymatic reaction kinetics; (2) drug-DNA/RNA interactions; (3) RNA folding; (4) protein-nucleic acid interactions; (5) nucleic acid-small molecule interactions; (6) nucleic acid-nucleic acid interactions; and (7) biomolecule-cell interactions. ITC is a method that can provide complete thermodynamic parameters of intermolecular interactions [118]. These parameters include binding constants, number of binding sites, molar enthalpy of binding, molar entropy of binding, molar constant-pressure heat capacity, and kinetic parameters.

Yu’s team rationally designed the DNA aptamer with a stem-loop structure, resulting in a substantial conformational change and retention of high affinity upon Cd^2+^ binding [119]. ITC characterization confirmed that the considerable entropy change indicated that the aptamer underwent a significant conformational change upon binding to the target. ITC enables the determination of multiple thermodynamic binding parameters in a single experiment. Stefen et al. [101] determined the dissociation constant characterizing aptamer binding by isothermal titration with a *K*_d_ value of 3.4 μM. Following the analysis, aptamers with high affinity and specificity were identified. Zhang et al. [120] constructed a microfluidic SELEX platform for efficient screening of late glycosylation end-product aptamers. The dissociation constant (*K*_d_) was obtained from isothermal titration analysis to be 6.65 ± 3.07 μM. Zhang et al. [121] employed ITC to characterize the magnesium dependence of the folding and binding mode of the fluorescent luminescent aptamer RhoBAST in solution and demonstrated its strong structural robustness. In the domain of food science, the Maillard reaction plays a pivotal role in the production of food color and flavor. A parallel can be drawn between the Maillard reaction and the microangiopathy that is characteristic of diabetes. The Maillard reaction is a complex chemical reaction between sugars and proteins that generates flavor substances [122]. The detection of glycosylated proteins, in combination with ITC, can be used to identify the optimal aptamer to bind with glycosylated proteins. This approach enables the analysis of protein glycation in real-time, facilitating intervention and regulation of the generation of flavor substances at an early stage. ITC technology faces significant challenges in accurately localizing ligand-target binding in complex systems.

The complete thermodynamic parameters (e.g., ΔH, ΔS) provided by ITC can directly quantify the mutation effect. For instance, Yu et al. [119] discovered that Cd^2+^ binding induces entropy-dominated conformational changes, which informed the design of highly sensitive electrochemical sensors. For extended applications of isothermal titration, microfluidic microarrays can be used in conjunction with ITC technology. These microarrays are highly sensitive and can detect very small sample amounts. They can also determine weak aptamer binding (*K*_d_ extends to less than mM) and analyze very low-concentration samples (less than μM) [120]. ITC technology can also be used to automate experiments by integrating a robotic arm and a multiple-well plate automated sampling system. This technology can be used for forward target screening, in which the aptamer is immobilized to identify targets with high affinity. It can also be used for reverse target screening, i.e., aptamer modification, target fixation, and the rapid search for optimal aptamer sequences. Therefore, it can be directly applied to aptamer optimization.

**Figure 7:**
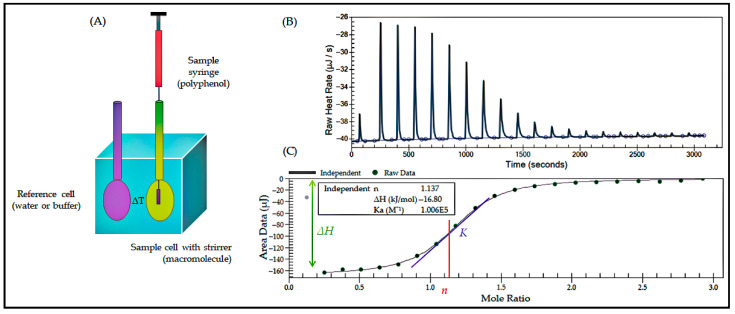
Isothermal titration calorimetry (ITC) technique related diagrams [123]. (**A**) The draft of isothermal titration calorimeter, (**B**) The exothermic interactions in the model thermogram, and (**C**) The sigmoidal binding isotherm fitted with a one-site model, where *Ka* = binding constant, *n* = number of binding sides, and *ΔH* = enthalpy change.

As illustrated in Figure 7, the isothermal titration technique is depicted in a simplified form.

#### 3.1.3. Microscale Thermophoresis

**Figure 8:**
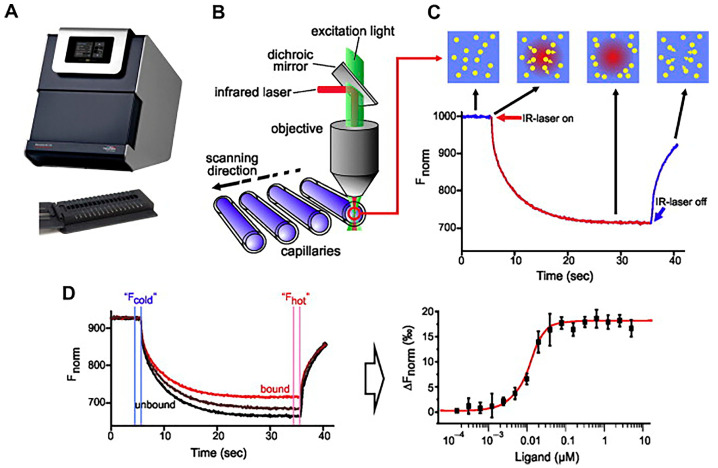
Microscale thermophoresis (MST) setup and experiments [128]. (**A**) The Monolith NT.115 from NanoTemper Technologies GmbH includes a capillary tray accommodating up to 16 capillaries. (**B**) This schematic illustrates the optical configuration for MST measurements: (1) Measurements use 4 μL capillaries, (2) A single objective handles both excitation and detection, (3) An infrared (IR) laser creates localized heating, (4) Fluorescent molecule movement is tracked along the temperature gradient. (**C**) Typical signal of an MST experiment: Initially, the molecules are homogeneously distributed, resulting in a constant “initial fluorescence” detection.Upon IR laser activation, within the first second, a “T-Jump” is observed, indicating a rapid change in fluorophore properties due to the temperature increase.Subsequently, thermophoresis leads to the movement of fluorescently labeled molecules away from the heated area. The fluorescence change is typically measured for 30 s. After IR laser deactivation, an inverse T-Jump occurs, followed by “back diffusion” of molecules, driven solely by mass diffusion, restoring the initial distribution gradually. (**D**) In a typical binding experiment, the thermophoretic movement of a fluorescent molecule (black trace, labeled “unbound”) undergoes alteration upon binding to a non-fluorescent ligand (red trace, labeled “bound”), leading to distinct traces. *ΔF*_norm_, representing the change in normalized fluorescence, is utilized for analysis. It is defined as *F*_hot_/*F*_cold_, where *F*-values correspond to the average fluorescence between areas marked by cursors. Titration of the non-fluorescent ligand causes a gradual shift in thermophoresis, plotted as *ΔF*_norm_ to produce a binding curve. This curve can be fitted to derive binding constants.

Microscale thermophoresis (MST) is a technique that is employed to analyze biomolecular interactions. The determination of affinity is achieved through the measurement of alterations in the hydration layer [124]. This technique enables the determination of various parameters, including the presence or absence of binding between molecules, the number of binding sites, and the affinity of intermolecular interactions [125]. MST typically involves the mixing of fluorescently labeled aptamers with varying concentrations of target solutions, followed by the use of MST instruments to measure the thermophoretic effect. Fluorescence signal changes are monitored by an infrared (IR) laser, which generates a temperature gradient within the capillary. By comparing the changes in fluorescence signals in different concentrations of target solutions, binding parameters such as dissociation constants (*K*_d_) are calculated [126]. These calculations, in turn, allow for a quantitative assessment of the binding affinity [127]. As illustrated in Figure 8, the working schematic of MST is depicted.

In the study by Liu et al. [129], the aptamer targeting function in relation to human plasma proteins was examined. Microscale thermophoresis was utilized to demonstrate that plasma interacts with the aptamer, thereby limiting the aptamer’s affinity for target tumor cells. The fluorophore position of the aptamer probe is critical for the MST response in aptamer-target binding, and the fluorophore type and labeling position also have a great influence on the MST of the aptamer probe. Yu et al. [130] analyzed the additive effect of aptamer fluorophore modifications on the microscale thermophoretic characterization of aptamer-target binding. This provides a basis for the rational design of aptamer probes to analyze aptamer-target interactions and for reliable affinity assays using MST. The MST aptamer sensor swiftly detects heavy metals in real water samples. In another study, Yu’s team developed a simple, fast, and sensitive aptamer MST sensor for the detection of Cd^2+^ [131]. The strategy was predicated on Cd^2+^ binding-induced changes in the MST information of the TMR-labeled aptamer.

MST, as a strategy that does not require immobilization and labeling measurements, is able to be highly sensitive to the thermal motion of molecules at weak temperature differences [132], allowing quantitative analysis of intermolecular interactions. The experimental procedures require minimal sample quantities, leading to reduced consumption [133]. Furthermore, the relative change in signaling is contingent on fluorophore modification of the aptamer, a process that is unfeasible in the absence of such modification. In addition, the MST technique is incapable of distinguishing between covalent binding, non-specific and specific binding, conformational changes, true binding, etc. The high sensitivity (pM-level) and low sample volume (μL-level) properties of MST make it suitable for precious sample optimization (e.g., rare nucleotide modification aptamers).

Moreover, Single-Molecule MST and High Throughput MST have been developed. Single-Molecule MST has the capacity to further reduce the detection limit, rendering it suitable for the identification of ultra-low abundance targets, such as rare transcription factors and exosome [134]. High-throughput MST is poised to enable large-scale screening of aptamer libraries. This approach will further facilitate investigation of the structure-activity relationship between structural modifications of a given aptamer and its target binding affinity [135].

#### 3.1.4. Fluorescence Analysis

Fluorescence analysis is a technique that utilizes the phenomenon of fluorescence to study the structure, dynamics, and interactions of nucleic acids as well as their function in biological processes. The binding of the aptamer to the target results in alterations in fluorescence anisotropy, thereby providing information regarding binding affinity and binding site [136]. Fluorescence anisotropy provides information about changes in molecular volume, structure, locally rotated fluorophores, or fluorescence lifetimes that affect FA/FP values [137]. The measurements of fluorescence anisotropy and polarization changes provide insight into molecular interactions, including binding affinity and binding sites. The field of fluorescence analysis is characterized by a concentration on the study of techniques, including, but not limited to, fluorescence resonance energy transfer (FRET) [138], fluorescent nucleic acid probes [139,140], and molecular beacons. The FRET technique has been demonstrated to be capable of identifying mutation sites (e.g., stem-zone rigidification) through distance sensitivity. However, the selection of donor-acceptor pairings has been observed to be a factor that can potentially obscure true conformational changes (e.g., ‘fluorescence quenching false positives’). The use of single-molecule FRET has been demonstrated as a means to monitor conformational changes in aptamers and to elucidate the kinetic pathways of aptamer-target binding [141]. The incorporation of nanomaterials, such as carbon dots [142] and noble metal nanomaterials [143], into the assay can enhance the fluorescence signal, thereby demonstrating the interaction of the substance to be tested with the aptamer at lower concentrations.

In the present study, fluorescence analysis has been implemented to expedite on-site detection. Wang et al. [144] developed a portable dual-mode fluorescence detection device to facilitate on-site detection in various set-tings. The developed dual-mode device has been shown to address this issue to a certain extent through ratio correction. For target molecules that exhibit fluorescent properties, the *K*_d_ value can be determined by detecting the change in fluorescence upon binding to the aptamer. As Liu et al. [42] demonstrated, fluorescence analysis can be utilized to assess aptamer affinity in aptamer in vitro screening and optimization [145]. Furthermore, the application of fluorescence analysis in the context of in vivo and in situ cellular imaging has emerged as a significant advancement in the field. The fluorescence-illuminated RNA adaptor (FLAP) has emerged as a promising tool for visualizing RNA in living cells [146]. In the domain of food analysis, it serves as a monitoring tool for the teratogenesis, carcinogenesis and mutagenesis of potentially harmful substances in food.

### 3.2. Computer Simulation Methods

#### 3.2.1. Molecular Docking

Molecular docking is a bioinformatics-based simulation method that evaluates the interactions between molecules and predicts their binding modes and affinities through a computerized platform. At present, a variety of software programs are available to study the binding ability and binding sites between aptamers and target molecules. Among the most commonly used software programs are Discovery Studio and AutoDock [147]. These two programs offer great functionality and flexibility. In circumstances involving more intricate systems of simulated docking, researchers have access to two additional software programs: GOLD and ICM [148]. The latter has demonstrated a high degree of accuracy in predicting fitness bonding and dealing with diverse targets.

Molecular docking is a common practice in agriculture, food safety, and healthcare, with applications including the description of detailed structures of aptamers, revelation of key binding regions of aptamers, the prediction of aptamer-target binding, the elucidation of molecular interactions, and the optimization of aptamer sequences [41,43,149,150]. Zhang et al. investigated the binding mechanisms and interaction points of targets and aptamers by CD and molecular docking to further guide the truncation and optimization of aptamers [120]. The advent of the digital age, accompanied by the continuous advancement of science and technology, has further promoted the development of data mining methodologies. Contemporary research in machine learning and artificial intelligence is progressively contributing to advancements in molecular docking algorithms. Zhao et al. [108] employed the program suite AutoDock 4.2.0 to perform molecular docking of the three dimensional structure of antiparallel quadruplex DNA. This was done to identify favorable binding conformations of the ligand at the target site.

Virtual screening of molecular docking has been demonstrated to accelerate the optimization cycle. For instance, Zhang et al. [151] improved the optimization efficiency of organophosphorus pesticide aptamers by 70%. However, the force field parameters are not sufficiently adapted to non-natural bases, which leads to >15% error in the calculation of binding energy [69]. Presently, the model must undergo iterative refinement, with its development contingent upon the analysis of experimental data. Meanwhile, the integration of molecular docking technology with artificial intelligence enables the utilization of the methodologies directly for physical modeling, thereby facilitating the expeditious attainment of docking outcomes [152]. The advent of artificial intelligence will precipitate a convergence of molecular docking and aptamer optimization, thereby facilitating the automated optimization of aptamer sequences and the automation of reverse target screening. Furthermore, as aptamers with rare bases attached are being used with increasing frequency, future docking methods will be employed for docking these aptamers as well.

#### 3.2.2. Molecular Dynamics

Molecular dynamics is a methodology that combines computational chemistry and biophysics to model the motions and interactions of atoms and molecules at a given time [118]. This method utilizes a numerical solution of the equations of motion of atoms or molecules to predict the dynamic behavior of a system. In contrast to molecular docking, molecular dynamics can dynamically demonstrate the process of target and aptamer binding. It does so by analyzing not only the final thermodynamic structure, such as docking energy and docking structure, but also the dynamics of molecular docking as a kinetic process. However, in comparison with molecular docking, the computational process of molecular dynamics is more intricate and time-consuming. Common molecular dynamics software includes GROMACS 2024, NAMD 3.0b3, and AMBER 22, among others.

Molecular dynamics has been demonstrated to serve a dual purpose in the field of aptamer research. Firstly, it enables the prediction of the final structure of aptamer-target binding and its stability. Secondly, it can be utilized to predict the kinetic process of aptamer-target molecular binding and reveal the binding mechanism in greater detail. Furthermore, molecular dynamics can serve as a reference point for the binding time of the aptamer and target in experimental settings. Ropii performed molecular dynamics simulations of six DNA aptamers that bind to cTnL proteins to elucidate their molecular interactions, founding that Tro4 exhibited the highest stability [149]. In his study of the structural mechanisms of RhoBAST binding and activation of contact burst fluorophores, Zhang demonstrated that highly heterogeneous conformational assemblies exhibit major contacts in both the free and bound TMR-DN [121]. In addition, the optimization of existing molecular force field parameters using machine learning algorithms that learn from large amounts of experimental data to achieve high-precision calculations. Ivanova et al. [153] developed Python-based tools that can simplify all stages of molecular dynamics simulations. Molecular Dynamics simulations have been demonstrated to reveal dynamic binding pathways (e.g., Ropii [149] discovered a mechanism of metastable activation of cTnL protein aptamers). However, ordinary simulations are computationally prohibitively expensive (about CPU months/system) for large aptamers (>80 nt). Solutions have also been proposed for coarse-grained models that reduce the computational load by 90% while retaining the key dynamics features and are more suitable for multiple rounds of optimization iterations [153].

As with molecular docking, molecular dynamics techniques can be directly combined with artificial intelligence to use the methods for physical modeling to obtain molecular dynamics results [154]. Moreover, as the use of aptamers with rare bases attached becomes increasingly prevalent, those methods will be employed for molecular dynamics of these aptamers. Quantum computing is now also gradually being used for molecular dynamics studies [155]. In the future, its use in molecular dynamics studies of aptamers and targets is likely to yield more accurate results in a shorter time. In summary, the experimental validation method of aptamer-target molecule binding ability in aptamer design is shown in Table 3.

## 4. Examples of the Application of Aptamer Sequence Optimization in Food Analysis

The optimization of aptamer sequences has been shown to offer a number of advantages, including high stability, high sensitivity, and low cost. These benefits have led to the widespread adoption of aptamer-based biosensors in fields such as food safety, bioanalysis, and environmental monitoring. These biosensors, which are constructed around nucleic acid aptamers, have a variety of detection mechanisms, including fluorescence, electrochemical, and Raman-enhanced spectroscopy. Optimized aptamers have been demonstrated to be effective in detecting pesticides, heavy metals, fungal toxins, pathogenic bacteria, and other objects.

### 4.1. Pesticide Detection

Presently, the misuse of pesticides has led to significant contamination of water bodies, soil, agricultural products, and food, among other consequences, which in turn have had indirect effects on human health [156]. The utilization of aptamer-based biosensors has become increasingly prevalent in recent years, owing to their ability to facilitate rapid detection of samples. In order to achieve the detection of pesticides with high sensitivity, some researchers have optimized the aptamers. Zhang et al. [151] developed a method to detect organophosphorus pesticides by recombinant broad-spectrum DNA aptamers that competitively bind to molecular beacons (MB) for quantitative measurement of pesticides. The aptamers selected by SELEX were structurally modified and truncated. Then the optimal aptamers were determined by molecular docking and kinetic simulation, with a limit of quantification as low as 13.4 nM in minced vegetable sample testing. Yang et al.’s team [157] performed high-throughput sequencing of the enriched ssDNA library by multiple rounds of Capture-SELEX. They utilized molecular docking to obtain a λ-triclopyr truncated aptamer with a *K*_d_ value of 10.27 ± 1.33 nM, as validated by MST. It demonstrated good repeatability and reproducibility in pear and cucumber samples. Aptamer optimization has been shown to enhance sensitivity, rendering it suitable for on-site testing of agricultural products in farmland, as well as water samples from lakes.

The optimization of aptamers can be enhanced through various modifications, including truncation, mutation, non-base modification, and the incorporation of rare bases. These modifications have the potential to improve the detection sensitivity of the aptamer. In the future, the aptamer can be optimized by attaching fluorescent groups or isotopes, so that it not only can bind to pesticides, but also can enter into cells and monitor the physiological effects of absorption, distribution, metabolism, and excretion of pesticides in cells through cellular imaging. This will lead to a more detailed exploration of pesticide toxicity mechanisms, toxicity prevention and control, and bacterial pesticide resistance mechanisms.

### 4.2. Heavy Metal Ions

Common heavy metal ions include Hg^2+^, Cd^2+^, As^3+^, Pb^2+^, and others. These heavy metals are commonly found in food products such as aquatic products, crops, and animal foods [158]. However, they pose a serious threat to ecosystems and human health due to their toxicity, persistence, and bioaccumulation. The ramifications of this phenomenon on environmental contamination and human wellbeing can be effectively mitigated through the implementation of sophisticated detection methodologies and regulatory interventions. A number of researchers have employed molecular dynamics simulation screening as a means to modify the nucleotides of Pb^2+^ aptamers, thereby engineering mutants that exhibit enhanced target affinity [159]. In addition to optimizing the length of the aptamer sequence, a research team found that altering the conformation of the aptamer can enhance the conformational stability of the heavy metal ion aptamer. Recently, Qian et al. [160] developed a locking aptamer probe in which Cd^2+^ was immobilized at the locking core position when bound to the probe, forming a stable cavity structure with a detection limit of 6.9 nM and a wide linear range. The spiked recovery rates of the aptamer sensors in shrimp and crab samples were 92.93–102.8%, respectively. The conformationally stable locked aptamer probe effectively enhances the stability of the aptamer-target complex and is suitable for the highly sensitive detection of Cd^2+^. The innovative application of aptamer optimization technology in the detection of heavy metal ions not only improves the detection performance, but also provides a new tool for environmental monitoring and food safety.

In the future work, the physiological effects of heavy metals on cellular uptake, distribution, metabolism, and excretion can be explored by combining the optimized aptamers with fluorescence and molecular imaging techniques. In particular, the molecular-level effects of heavy metals on various cellular organelles (particularly the nucleus and mitochondria) can be investigated. Therapeutic methods can be proposed, such as the use of a drug’s heavy metal binding capacity. This capacity is characterized by a competition between the heavy metal and the aptamer for the same binding site. Once successful, the result is the disappearance of the fluorescence signal. Moreover, recent literature has documented the emergence of drug-resistant, pathogenic superbugs in heavily polluted areas, a phenomenon that has been attributed to the presence of heavy metals [161]. This development poses a grave threat to agriculture and food security. Consequently, the employment of heavy metal aptamers, which possess the capacity to penetrate bacterial membranes, in conjunction with this methodology, offers a promising avenue for elucidating the underlying mechanisms of heavy metal-induced bacterial mutation. This approach may serve as a crucial step in the mitigation of the emergence of such superbugs.

### 4.3. Fungal Toxins

Mycotoxins are toxic secondary metabolites produced by certain fungi during their growth process [162]. These toxins are widely present in food, feed, and the environment, posing a serious threat to human and animal health. Mycotoxins possess significant toxicity, including aflatoxin, ochracin, zearalenone, trichothecin, fumonisin, patulin, citrinin, and clostridium perfingen. In response to this toxicity, researchers have developed a range of novel detection methods. Among these methods, aptamer biosensors have gained widespread application.

Furthermore, Guan’s team [163] developed a novel label-free chemiluminescent assay for zearalenone based on a truncated aptamer that binds to G-quadruplex DNAzyme. In this particular instance, the majority of the key parameters of the truncated aptamer were optimized through molecular docking analysis with the objective of maximizing sensitivity. The final achievement was to demonstrate perfect linearity (R^2^ = 0.9996) for zearalenone detection over a concentration range of 3.14–314.10 nM within 40 min, reaching a detection limit of 8.95 nM, a 6.7-fold improvement over the pre-optimization period. The aptasensor obtained a satisfactory recovery rate of 92.84–137.27% and 84.90–124.24% for ZEN-spiked wheat and maize samples, respectively. In regard to aflatoxin, certain researchers have undertaken a rational and systematic optimization of the AFB1 luminescent aptamer through a computer-guided engineering strategy guided by a computer simulation. The initial 80-nucleotide (nt) AFB1 aptamer was truncated to obtain the minimal active structure of 13 nt. The construction of the label-free ratio luminescent aptamer sensor for sensitive on-site detection of AFB1 was then accomplished, allowing for the detection of AFB1 in a mere three minutes with a detection limit as low as 36 ng/L [164]. Concurrently, aptamer optimization technology has the potential to generate novel concepts and methodologies for the identification of novel mycotoxins, thereby facilitating the continual improvement of mycotoxin detection systems in food.

In the future, optimizing mycotoxin-specific aptamers alongside those targeting other hazardous substances, combined with molecular imaging techniques, will enable in-depth investigation of the synergistic deleterious effects of mycotoxins and other toxins on human cells [165]. For instance, this approach could elucidate the synergistic mechanisms of aflatoxin with hepatitis B virus, alcohol, and other hepatotoxic agents in driving liver cirrhosis and hepatocellular carcinoma [166]. Future studies may explore synergies such as: (1) Ochratoxin and nephrotoxic plant toxins (e.g., aristolochic acid) on renal damage; (2) Zearalenone interacting with sex hormones, polyphenols, and/or other endocrine disruptors on reproductive organs. Furthermore, integration with High-Throughput Cytotoxicity Screening Platforms will identify novel target cells for mycotoxin toxicity. This strategy accelerates target organ identification and toxicity profiling, ultimately improving mycotoxin hazard prevention and control.

### 4.4. Pathogenic Bacteria

Pathogenic bacteria are microorganisms that can cause disease, are widely distributed in life, and usually enter the human body through food, thus posing a threat to human health. The process of quantification and characterization of pathogenic bacteria faces challenges such as time-consuming and low sensitivity [167]. Yang et al. [168] developed a magnetic separation-assisted DNAzyme-driven 3D DNA walker fluorescent aptamer sensor for the detection of the foodborne pathogen Cronobacter spp. This sensor was created by optimizing the original aptamer sequence by a truncation strategy. The novel method exhibits a detection limit as low as 2 CFU/mL, enabling the ultra-sensitive and selective detection of Cronobacter spp. This method has good spiked recovery rates in water, infant formula, ice cream, and milk samples. In addition, Sun et al. [169] developed a hybridization chain reaction (HCR)-based multivalent aptamer (Multi-Apt) signal amplifier for high-sensitivity detection of Salmonella. The assembly of the aptamer on the HCR product resulted in the construction of a DNA-programmable Multi-Apt, which exhibited a binding affinity 33-fold higher than that of the monovalent aptamer (Mono-Apt). The detection limits for milk, egg whites, and chicken meat were calculated to be 128, 185, and 164 CFU/mL, respectively. Presently, bacterial aptamer sensors demonstrate considerable superiority over conventional detection methodologies with regard to sensitivity, operational simplicity, and cost management. In the future, the optimization of aptamer selection, the perfection of sensor construction, and the application in the actual environment need to be further solved, so as to enhance its application value and market potential, and to guarantee food safety [170].

In aptamer optimization, non-base modifications enable rare base technology applications for monitoring cancer cell drug resistance [171]. This approach holds promise for early detection of drug-resistant and highly pathogenic bacteria, allowing eradication before severe drug resistance and enhanced pathogenicity emerge. Such prevention mitigates superbug evolution and curbs drug-resistant bacterial proliferation.

Integrated aptamer optimization and molecular imaging technologies have enabled tumor monitoring [76]. These techniques can similarly track bacterial metabolism and physiology. This synergy advances include chemical disinfection (developing non-toxic bactericides) and physical sterilization (revealing molecular mechanisms for low-dose efficacy).

Aptamer-based anti-tumor agents demonstrate dual detection/therapeutic functionality with minimal human toxicity [172]. Similarly, developing dual-functional aptamers for simultaneous pathogen detection and sterilization is feasible. Optimized for biosafety, these aptamers offer human-compatible solutions for food safety applications.

### 4.5. Others

In addition to pesticides, heavy metal ions, fungal toxins, and pathogenic bacteria, other substances, including perfluorooctanoic acid (PFOA), bisphenols, and bacterial toxins, have also been demonstrated to be harmful to the human body. Biosensors that have been optimized based on aptamers can demonstrate equivalent efficiency in detecting these substances. The structural features of heat-resistant direct hemolysin (TDH) have been thoroughly examined, and a stable multivalent aptamer has been rationally designed by truncating the key fragment evolution and end fixation. This optimized aptamer has been demonstrated to be useful not only for the detection of TDH, but also for showing a strong potential to counteract the toxicity of TDH by inhibiting the formation of membrane pores. This provides a new method to mitigate bacterial infection [173]. Bisphenol A (BPA) is a chemical compound that has been demonstrated to have an impact on human health [174]. It is a constituent of numerous packaging materials and has the potential to migrate into the human body through contact with foodstuffs. A previous research team designed a high-affinity aptamer for its ultra-sensitive colorimetric detection, and the detection limit was as low as 7.60 pM, which is 265 times lower than that using conventional aptamers [175]. Subsequent studies have employed base mutation motif-targeted aptamers to systematically analyze bisphenol analogs (BPs) in complex water, leveraging molecular docking calculations to identify group-targeted aptamers capable of binding to BPs, with a detection limit as low as 6.7 pM [176]. Furthermore, antibiotics are commonly found in food products.

The optimized aptamer demonstrates notable superiority in the detection of antibiotics. Aissa et al. [177] conducted a rapid detection study of oxfloxacin (OFL) employing truncated aptamers of Fluorescent Resonance Energy Transfer (FRET). The truncated aptamers exhibited higher specificity for OFL in comparison to other quinolones, as evidenced by a previous study that utilized full-length aptamers. Guo et al. [178] obtained optimized truncated aptamers of Spreading Penicillin based on the truncation method of aptamer secondary structure and verified it by molecular docking and CD technology. They then used aptamers modified with gold nanoclusters (AuNCs) as a fluorescent probe and a strategy that involved DNase I-assisted fluorescence signal amplification. This strategy successfully improved the sensitivity of the aptamer sensor. The developed assay exhibited a low detection limit of 8.5 ng/L, and can be effectively used in apple and grape juice samples. It has good practical application in milk with added standard samples. Sun et al. [179] constructed an ultra-stable fluorescent aptamer sensor based on MAPbBr3@ZIF-8 for the highly sensitive and specific detection of alloxacillin (AzL) in food products by truncating to obtain the aptamer with the highest affinity and a detection limit of 25 ng/L. The design integrates the advantageous optical characteristics of chalcogenides, capacity for rapid fluorescence enhancement exhibited by AuNCs, and the specificity characteristic of aptamers. This integration suggests considerable potential for the analysis of Azl in food samples.

In summary, aptamer optimization is a widely utilized technique in biosensor-based detection methods in food safety analysis and other fields. Among these, truncation has emerged as the predominant strategy for aptamer optimization, owing to its efficient and superior characteristics. In signal amplification systems, the employment of optimized aptamers has been demonstrated to enhance sensitivity, stability, and affinity. The incorporation of nanomaterials as signal amplification elements has also been observed to facilitate the formation of a comprehensive system. Following aptamer sequence optimization, a general reduction in the detection limit was observed, along with a significant decrease in reagent consumption and enhancement of stability in complex samples. The aptamer sequence optimization strategy is a subject of significant research value, as evidenced by these substantial advantages. As illustrated in Table 4, recent literature has documented a range of strategies employed for aptamer optimization.

## 5. Conclusions

This paper provides a synopsis of several frequently employed aptamer sequence optimization strategies and their aptamer applications in food safety analysis. Additionally, it offers an overview of optimization metrics, including molecular docking, molecular dynamics, circular dichroism, fluorescence analysis, and other techniques.

In recent years, there has been a gradual increase in the incorporation of non-natural nucleotides into aptamer sequences. It is anticipated that the future development of aptamers will involve the application of more heterocycles and modifiers. Furthermore, the application of metal-organic heterocyclic compounds in the construction of aptamers has emerged as a significant area of research. The development of these aptamers has led to the prospect of enhanced detection capabilities and therapeutic interventions.

Concurrently, artificial intelligence and big data analytics technologies are assuming an increasingly prominent role in the discovery and optimization of nucleic acid aptamers. At present, the majority of data mining research is oriented towards mechanistic explanations. The potential for data mining techniques to be applied to aptamer sequence optimization and more complex systems of action in the future is significant. This development has the potential to stimulate aptamer research. High-throughput sequencing technology produces a considerable amount of data that remains underutilized. The development of advanced algorithms for the processing and analysis of this data to enhance its utilization is poised to emerge as a prominent trend in aptamer research in the future.

Furthermore, research on aptamer optimization should prioritize cross-field applications, particularly the design and optimization of aptamers in complex biological systems in food. This will offer novel concepts for the extensive application of aptamer technology. The following areas of research are of particular importance: the breeding of food-borne animals in the food field, the prevention of epidemics, and the promotion of health; the early identification of pathogenic microorganisms in food species; the analysis of drug resistance; and the rapid detection of new contaminants. In addition, the design of the aptamer sensors should prioritize the enhancement of intelligence, portability, and real-time monitoring capability to meet the detection needs in different scenarios, such as food processing and storage. In conclusion, with the continuous progress of artificial intelligence and various technical aids, their role in high-throughput sequence analysis and identification of nucleic acid aptamers, and sequence optimization and design are becoming more and more prominent in order to ensure food quality and safety.

## Figures and Tables

**Table 1:** Characterization of sugar structures in different nucleic acids.

Nucleic Acid Types	Ribose Chemical Structure	Ribose Structural Features	Nucleic Acid’s Key Features
DNA	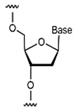	Contains 2′-deoxyribose and lacks 2′-hydroxyl group.	Highly stable, high affinity, high specificity.
RNA	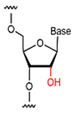	The sugar ring structure is ribose with a hydroxyl group at the 2′ position.	Relatively unstable and prone to degradation.
LNA	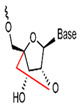	Positions 2′ and 4′ are connected by a methyl bridge to form a rigid structure.	High affinity, high specificity, resistance to nuclease degradation, single-base mismatch discrimination.
PNA	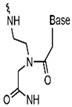	Peptide backbone, sugar-phosphate backbone replaced by nonionic, repeating N-(2-aminoethyl)glycine units.	Resistant to nuclease and protease degradation, high thermal stability, single base mismatch identification, can be used for nucleic acid sensing.
TNA	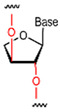	2′, 3′-linked sugar ring structure with acid stabilization.	High affinity, high specificity, resistant to nuclease degradation, able to stabilize in vivo.
HNA	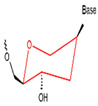	It contains a six-carbon sugar ring for high stability and resistance to nuclease degradation.	High affinity, high specificity, able to stabilize in the physiological environment.
CeNA	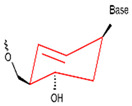	It contains a cyclohexenose ring for high stability and resistance to nuclease degradation.	High thermal stability, RNase H activation, single base mismatch identification ability.
GNA	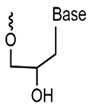	It contains a glycol backbone for high stability and resistance to nuclease degradation.	High thermal stability, single base mismatch identification ability, can be used for nucleic acid sensing, gene detection.
F-ANA	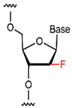	2′ position contains a fluorine modification for high stability and resistance to nuclease degradation.	High affinity, high specificity, able to stabilize in physiological environment.
ANA	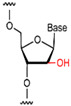	2′ position contains a hydroxyl group, which is resistant to nuclease degradation.	High affinity for stabilization in the physiological environment.

**Table 2:** Characteristics of different heterocycles (non-natural bases).

Heterocyclic Name	Heterocyclic Structure	Chemical Structure Characteristics	Application Potential
D5SICS	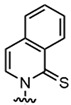	Isoquinolone backbone, hydrophobic interactions	Expanding the Genetic Code, Biotechnology Applications
DMMO2	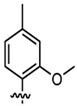	Benzimidazole backbone, hydrophobic interactions	Expanding the Genetic Code, Biotechnology Applications
P	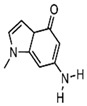	Exhibits high fidelity in Taq DNA polymerase-mediated PCR	qPCR assay, molecular beacon technique
Z	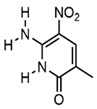	Nitro group for improved chemical stability	RNA labeling, dynamic structure analysis
NaM	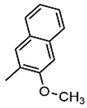	Naphthylimidazole backbone, hydrophobic interactions	Creating Semi-Synthetic Organisms (SSOs)
5SICS	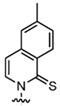	Isoquinolone backbone, hydrophobic interactions	Creating Semi-Synthetic Organisms (SSOs)
Ds	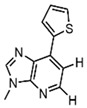	Dibenzocycloheptane skeleton, hydrophobic interactions	RNA labeling, dynamic structure analysis
Px	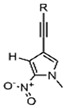	Pyridine backbone, hydrophobic interactions	RNA labeling, dynamic structure analysis

**Table 3:** Experimental validation methods for aptamer-target molecule binding capacity in aptamer design.

Validation Methods	Principle	Core Advantages	Application Scenarios
Molecular docking	Predicting the binding mode and binding energy between two molecules.	Rapid prediction of molecular binding sites and thermodynamic analysis of docking feasibility.	Food packaging material design and development, food toxin detection and toxicology research, target and aptamer docking.
Molecular dynamics	Analyzing the behavior of molecules through mathematical models and computer simulation techniques.	Dynamics reveals the interaction mechanism between the aptamer and the target molecule, and it can indicate the rate of docking. The feasibility of docking is analyzed from thermodynamic and kinetic perspectives.	Process guidance and optimization in food processing, aptamer stability studies, DNA structure modeling, intermolecular interaction analysis.
Circular dichroism	Study of optical activity based mainly on differences in the absorption of polarized light by chiral centers in molecules.	Provides secondary structure information and unlabeled structure analysis.	Structural molecules of biomolecules, secondary structure studies of aptamers, and DNA structural stability analysis.
Isothermal titration	Study of intermolecular interactions by heat differences measured under isothermal conditions.	Provide thermodynamic parameters, high sensitivity, automation, and high accuracy.	Molecular interactions, aptamer-target molecule affinity determination, and food safety analysis.
Microscale thermophoresis	Measurement of trace thermophoretic changes to determine intermolecular affinity by changes in the hydration layer.	Broad compatibility, low sample consumption, and low running costs.	Aptamer affinity, molecular dynamics and molecular physicochemical property studies.
Fluorescence analysis	Based on the fluorescence of a substance when exposed to light at a specific wavelength.	High sensitivity, high selectivity, low sample volume, simple operation.	Biomedical, food safety, aptamer-target molecule interaction studies, and intermolecular interactions.

**Table 4:** Aptamer optimization strategies reported in recent years.

Target Classification	Sequence Optimization Methods	Application and Optimized Features	References
Zearalenone	Truncation	Food safety analysis. Increase sensitivity.	[180]
Chloromycetin	Truncation	Food safety control. High affinity and low synthesis cost.	[181]
Streptomycin	Truncation	Food safety control. High affinity	[182]
Organic Phosphorus pesticides	Truncation	Food safety analysis. Excellent recognition performance, high stability.	[41]
Deoxynivalenol	Truncation	Food safety analysis. High affinity and low cost.	[43]
Sulfaquinoxaline	Truncation	Environment and food safety. High affinity and sensitivity.	[183]
α-lactalbumin	Truncation	Food safety and medical diagnostics. Increased bonding affinity.	[42]
PTK7	Mutation	Diagnosis of disease treatment. Improved thermal stability and binding affinity.	[184]
Nucleic acid group	Mutation	Treatment and diagnosis. Improved nuclease resistance and affinity.	[51]
Kanamycin	Mutation	Food safety analysis. Increased sensitivity.	[185]
Vibrio parahaemolyticus	Mutation	Food safety control. Decreased affinity.	[52]
SARS-CoV-2	Mutation	Treatment and diagnosis. Enhanced viral inhibition.	[186]
FTO	Chemical modification	Clinical diagnosis, drug discovery and therapeutic evaluation. Reference for medical imaging analysis.	[68]
Thrombin	Chemical modification	Medicine and biotechnology. Enhanced stability.	[69]
Cytosine	Chemical modification	Research, diagnosis and treatment. Enhanced stability and rapport.	[187]
Uracil	Chemical modification	Treatment and diagnosis. Enhanced stability and rapport.	[188]
Quinine	Chemical modification	Disease treatment. Enhanced bonding affinity.	[189]

## Data Availability

The datasets generated during the current study are available from the corresponding author on reasonable request.

## References

[1] Duan N., Gong W., Wu S., Wang Z. (2017). Selection and Application of ssDNA Aptamers against Clenbuterol Hydrochloride Based on ssDNA Library Immobilized SELEX. J. Agric. Food Chem..

[2] Seok Kim Y., Ahmad Raston N.H., Bock Gu M. (2016). Aptamer-Based Nanobiosensors. Biosens. Bioelectron..

[3] Guo Z., Gao L., Jiang S., El-Seedi H.R., El-Garawani I.M., Zou X. (2023). Sensitive Determination of Patulin by Aptamer Functionalized Magnetic Surface Enhanced Raman Spectroscopy (SERS) Sensor. J. Food Compos. Anal..

[4] Wu S., Liu L., Duan N., Li Q., Zhou Y., Wang Z. (2018). Aptamer-Based Lateral Flow Test Strip for Rapid Detection of Zearalenone in Corn Samples. J. Agric. Food Chem..

[5] Luo L., Liu X., Ma S., Li L., You T. (2020). Quantification of Zearalenone in Mildewing Cereal Crops Using an Innovative Photoelectrochemical Aptamer Sensing Strategy Based on ZnO-NGQDs Composites. Food Chem..

[6] Wu X., Yin L., Gao S., Zhou R., Zhang Y., Xue S., Jayan H., El-Seedi H.R., Zou X., Guo Z. (2024). Core-Satellite Nanoassembly System with Aptamer-Conjugated Au@Ag Nanoparticles for SERS Detection of Patulin in Apples. Food Control.

[7] Peinetti A.S., Lake R.J., Cong W., Cooper L., Wu Y., Ma Y., Pawel G.T., Toimil-Molares M.E., Trautmann C., Rong L. (2021). Direct Detection of Human Adenovirus or SARS-CoV-2 with Ability to Inform Infectivity Using DNA Aptamer-Nanopore Sensors. Sci. Adv..

[8] Li H., Ahmad W., Rong Y., Chen Q., Zuo M., Ouyang Q., Guo Z. (2020). Designing an Aptamer Based Magnetic and Upconversion Nanoparticles Conjugated Fluorescence Sensor for Screening Escherichia Coli in Food. Food Control.

[9] Liu R., Zhang Y., Ali S., Haruna S.A., He P., Li H., Ouyang Q., Chen Q. (2021). Development of a Fluorescence Aptasensor for Rapid and Sensitive Detection of Listeria Monocytogenes in Food. Food Control.

[10] Li H., Bei Q., Zhang W., Marimuthu M., Hassan M.M., Haruna S.A., Chen Q. (2023). Ultrasensitive Fluorescence Sensor for Hg^2+^ in Food Based on Three-Dimensional Upconversion Nanoclusters and Aptamer-Modulated Thymine-Hg^2+^-Thymine Strategy. Food Chem..

[11] Guo Z., Chen P., Yin L., Zuo M., Chen Q., El-Seedi H.R., Zou X. (2022). Determination of Lead in Food by Surface-Enhanced Raman Spectroscopy with Aptamer Regulating Gold Nanoparticles Reduction. Food Control.

[12] Song S.-H., Gao Z.-F., Guo X., Chen G.-H. (2019). Aptamer-Based Detection Methodology Studies in Food Safety. Food Anal. Methods.

[13] Liu S., Meng S., Wang M., Li W., Dong N., Liu D., Li Y., You T. (2023). In-Depth Interpretation of Aptamer-Based Sensing on Electrode: Dual-Mode Electrochemical-Photoelectrochemical Sensor for the Ratiometric Detection of Patulin. Food Chem..

[14] Liu R., Ali S., Haruna S.A., Ouyang Q., Li H., Chen Q. (2022). Development of a Fluorescence Sensing Platform for Specific and Sensitive Detection of Pathogenic Bacteria in Food Samples. Food Control.

[15] Sharma A.S., Ali S., Sabarinathan D., Murugavelu M., Li H., Chen Q. (2021). Recent Progress on Graphene Quantum Dots-based Fluorescence Sensors for Food Safety and Quality Assessment Applications. Comp. Rev. Food Sci. Food Safe.

[16] Bi X., Li L., Luo L., Liu X., Li J., You T. (2022). A Ratiometric Fluorescence Aptasensor Based on Photoinduced Electron Transfer from CdTe QDs to WS2 NTs for the Sensitive Detection of Zearalenone in Cereal Crops. Food Chem..

[17] Zhang X., Zhou Y., Huang X., Hu X., Huang X., Yin L., Huang Q., Wen Y., Li B., Shi J. (2023). Switchable Aptamer-Fueled Colorimetric Sensing toward Agricultural Fipronil Exposure Sensitized with Affiliative Metal-Organic Framework. Food Chem..

[18] Li N., Ren C., Hu Q., Wang B., Yang Z., Xiao L., Guan T. (2025). Multiplex Aptamer Cluster Detection Platform and Systems Toxicology Study for 17β-Estradiol, Bisphenol A, and Diethylstilbestrol. Food Chem..

[19] Li L., Ma R., Wang W., Zhang L., Li J., Eltzov E., Wang S., Mao X. (2023). Group-Targeting Aptamers and Aptasensors for Simultaneous Identification of Multiple Targets in Foods. TrAC Trends Anal. Chem..

[20] Li W., Pei Y., Wang J. (2022). Development and Analysis of a Novel AF11–2 Aptamer Capable of Enhancing the Fluorescence of Aflatoxin B1. Chin. Chem. Lett..

[21] Gao J., Liu N., Zhang X., Yang E., Song Y., Zhang J., Han Q. (2022). Utilizing the DNA Aptamer to Determine Lethal α-Amanitin in Mushroom Samples and Urine by Magnetic Bead-ELISA (MELISA). Molecules.

[22] Wang J., Li X., Lei H., Liu J. (2024). Selection of DNA Aptamers for Detecting Metronidazole and Ibuprofen: Two Common Additives in Soft Drinks. Analyst.

[23] Qin Y., Shen J., Qin Y., Hayilati B., Yao J., Zhang M. (2024). Research Progress on the Application of Aptamer Optimization Technology and Portable Sensing Devices in Food Safety Detection. Crit. Rev. Food Sci. Nutr..

[24] Li R., Zhu Q., Sun X., Li Z., Liu X. (2024). Electrochemical Biosensor Based on the Integration of Maple Leaf-like Gold Nanocrystal and Truncated Aptamer for Detection of α-Amanitin with High Sensitivity, Selectivity and Rapidity. Food Chem..

[25] Debiais M., Lelievre A., Smietana M., Müller S. (2020). Splitting Aptamers and Nucleic Acid Enzymes for the Development of Advanced Biosensors. Nucleic Acids Res..

[26] Huang T., Chen X., Chen J., Zhang Y., Wang X., Wu Z., Zhou N. (2024). In Vitro Selection and Engineering Azithromycin-Specific Aptamers and Construction of a Ratiometric Fluorescent Aptasensor for Sensitive Detection of Azithromycin. Sens. Actuators B Chem..

[27] Hu X., Tang L., Zheng M., Liu J., Zhang Z., Li Z., Yang Q., Xiang S., Fang L., Ren Q. (2022). Structure-Guided Designing Pre-Organization in Bivalent Aptamers. J. Am. Chem. Soc..

[28] Ji D., Feng H., Liew S.W., Kwok C.K. (2023). Modified Nucleic Acid Aptamers: Development, Characterization, and Biological Applications. Trends Biotechnol..

[29] Alkhamis O., Canoura J., Ly P.T., Xiao Y. (2023). Using Exonucleases for Aptamer Characterization, Engineering, and Sensing. Acc. Chem. Res..

[30] Thevendran R., Citartan M. (2022). Assays to Estimate the Binding Affinity of Aptamers. Talanta.

[31] Manceau M., Farre C., Lagarde F., Mathey R., Buhot A., Vidic J., Léguillier V., Hou Y., Chaix C. (2024). Investigation of the Affinity of Aptamers for Bacteria by Surface Plasmon Resonance Imaging Using Nanosomes. ACS Appl. Mater. Interfaces.

[32] Li M., Qiu Y., Liu G., Xiao Y., Tian Y., Fang S. (2024). Plasmonic Colorimetry and G-Quadruplex Fluorescence-Based Aptasensor: A Dual-Mode, Protein-Free and Label-Free Detection for OTA. Food Chem..

[33] Écija-Arenas Á., Kirchner E.-M., Hirsch T., Fernández-Romero J.M. (2021). Development of an Aptamer-Based SPR-Biosensor for the Determination of Kanamycin Residues in Foods. Anal. Chim. Acta.

[34] Radi A.-E., Abd-Ellatief M.R. (2021). Electrochemical Aptasensors: Current Status and Future Perspectives. Diagnostics.

[35] Formen J.S.S.K., Howard J.R., Anslyn E.V., Wolf C. (2024). Circular Dichroism Sensing: Strategies and Applications. Angew. Chem. Int. Ed..

[36] Bastos M., Abian O., Johnson C.M., Ferreira-da-Silva F., Vega S., Jimenez-Alesanco A., Ortega-Alarcon D., Velazquez-Campoy A. (2023). Isothermal Titration Calorimetry. Nat. Rev. Methods Primers.

[37] Stein J.A.C., Ianeselli A., Braun D. (2021). Kinetic Microscale Thermophoresis for Simultaneous Measurement of Binding Affinity and Kinetics. Angew. Chem. Int. Ed..

[38] Xu Y., Huang T., Wang S., Yan Y. (2022). Mesoporous Silica-Based Molecularly Imprinted Fluorescence Sensor for the Ultrafast and Sensitive Recognition of Oxytetracycline. J. Food Compos. Anal..

[39] Perrier S., Bouilloud P., De Oliveira Coelho G., Henry M., Peyrin E. (2016). Small Molecule Aptamer Assays Based on Fluorescence Anisotropy Signal-Enhancer Oligonucleotides. Biosens. Bioelectron..

[40] Wang S., Ma R., Li L., Wang L., Li J., Sun J., Mao X., Tan W. (2022). Engineering Robust Aptamers with High Affinity by Key Fragment Evolution and Terminal Fixation. Anal. Chem..

[41] Chen P., Hu C., Tao X., Zhou Z., Wang L., Yang X., Che Z., Chen X., Huang Y. (2023). Recognition Mechanism and Sequence Optimization of Organophosphorus Pesticides Aptamers for Better Monitoring Contaminations in Food. Food Sci. Hum. Wellness.

[42] Liu R., Zhang F., Shi M., Sang Y., Wang X. (2022). In Vitro Selection and Optimization of High-Affinity Aptamer for Milk Allergen α-Lactalbumin and Its Application in Dual-Mode Detection. Front. Nutr..

[43] Han X., Qin M., Lu X., Wang Q., Zhang Y., Wang Z. (2025). A Highly Sensitive SERS Aptasensor Using a Novel Truncated Aptamer for the Detection of Deoxynivalenol. Sens. Actuators B Chem..

[44] Jiang H., Qi S., Khan I.M., Dong X., Wang Z., Yang J. (2024). CRISPR-Cas12a-Mediated Colorimetric Aptasensor of Netilmicin Based on Enzymes-Assisted Signal Amplification and Nanozyme Employing a Rationally Truncated Aptamer. Sens. Actuators B Chem..

[45] Yu Y., Chen K., Du Z., Fang B., Zhan J., Zhu L., Xu W. (2024). Magnetic Aptamer Copper Nanoclusters Fluorescent Biosensor for the Visual Detection of Zearalenone Based on Docking-Aided Rational Tailoring. Food Chem..

[46] Nguyen M.-D., Osborne M.T., Prevot G.T., Churcher Z.R., Johnson P.E., Simine L., Dauphin-Ducharme P. (2024). Truncations and in Silico Docking to Enhance the Analytical Response of Aptamer-Based Biosensors. Biosens. Bioelectron..

[47] Chen J., He J., Bing T., Feng Y., Lyu Y., Lei M., Tan W. (2024). Identification of the Binding Site between Aptamer Sgc8c and PTK7. Anal. Chem..

[48] Gao L., Zhang Y., Chen L., Zhou Q., Zhou N., Xia X. (2024). Study of Dual Binding Specificity of Aptamer to Ochratoxin A and Norfloxacin and the Development of Fluorescent Aptasensor in Milk Detection. Talanta.

[49] Li X., Chen X., Mao M., Peng C., Wang Z. (2022). Accelerated CRISPR/Cas12a-Based Small Molecule Detection Using Bivalent Aptamer. Biosens. Bioelectron..

[50] Wang S., Liu X., Wei D., Zhou H., Zhu J., Yu Q., Luo L., Dai X., Jiang Y., Yu L. (2024). Polyvalent Aptamer Nanodrug Conjugates Enable Efficient Tumor Cuproptosis Therapy Through Copper Overload and Glutathione Depletion. J. Am. Chem. Soc..

[51] Zhou J., Rossi J. (2017). Erratum: Aptamers as Targeted Therapeutics: Current Potential and Challenges. Nat. Rev. Drug Discov..

[52] Sun Y., Duan N., Ma P., Liang Y., Zhu X., Wang Z. (2019). Colorimetric Aptasensor Based on Truncated Aptamer and Trivalent DNAzyme for *Vibrio Parahemolyticus* Determination. J. Agric. Food Chem..

[53] Chen H., Li Y., Xiao Z., Li J., Li T., Wang Z., Liu Y., Tan W. (2023). Chemical Amplification-Enabled Topological Modification of Nucleic Acid Aptamers for Enhanced Cancer-Targeted Theranostics. ACS Nano.

[54] Troisi R., Riccardi C., Pérez De Carvasal K., Smietana M., Morvan F., Del Vecchio P., Montesarchio D., Sica F. (2022). A Terminal Functionalization Strategy Reveals Unusual Binding Abilities of Anti-Thrombin Anticoagulant Aptamers. Mol. Ther. Nucleic Acids.

[55] Das G., Harikrishna S., Gore K.R. (2022). Influence of Sugar Modifications on the Nucleoside Conformation and Oligonucleotide Stability: A Critical Review. Chem. Rec..

[56] Yu H., Zhao Q. (2022). A Sensitive Aptamer Fluorescence Anisotropy Sensor for Cd2+ Using Affinity-Enhanced Aptamers with Phosphorothioate Modification. Biosensors.

[57] Mana T., Bhattacharya B., Lahiri H., Mukhopadhyay R. (2022). XNAs: A Troubleshooter for Nucleic Acid Sensing. ACS Omega.

[58] Gutfreund C., Betz K., Abramov M., Coosemans F., Holliger P., Herdewijn P., Marx A. (2025). Structural Insights into a DNA Polymerase Reading the Xeno Nucleic Acid HNA. Nucleic Acids Res..

[59] Alves Ferreira-Bravo I., DeStefano J.J. (2021). Xeno-Nucleic Acid (XNA) 2’-Fluoro-Arabino Nucleic Acid (FANA) Aptamers to the Receptor-Binding Domain of SARS-CoV-2 S Protein Block ACE2 Binding. Viruses.

[60] Sin M.L., Mach K.E., Wong P.K., Liao J.C. (2014). Advances and Challenges in Biosensor-Based Diagnosis of Infectious Diseases. Expert. Rev. Mol. Diagn..

[61] Wang X., Yu D., Chen L. (2023). Antimicrobial Resistance and Mechanisms of Epigenetic Regulation. Front. Cell. Infect. Microbiol..

[62] Rangel A.E., Chen Z., Ayele T.M., Heemstra J.M. (2018). In Vitro Selection of an XNA Aptamer Capable of Small-Molecule Recognition. Nucleic Acids Res..

[63] Palme D.I., Lang J., Helke D., Kuryshko M., Abdelwhab E.M. (2024). Strain-Dependent Variations in Replication of European Clade 2.3.4.4b Influenza A(H5N1) Viruses in Bovine Cells and Thermal Inactivation in Semi-Skimmed or Whole Milk. Eurosurveillance.

[64] Raymond P., Paul S., Perron A., Deschênes L., Hara K. (2021). Extraction of Human Noroviruses from Leafy Greens and Fresh Herbs Using Magnetic Silica Beads. Food Microbiol..

[65] Nigam D., LaTourrette K., Souza P.F.N., Garcia-Ruiz H. (2019). Genome-Wide Variation in Potyviruses. Front. Plant Sci..

[66] Chen Z., Luo H., Gubu A., Yu S., Zhang H., Dai H., Zhang Y., Zhang B., Ma Y., Lu A. (2023). Chemically Modified Aptamers for Improving Binding Affinity to the Target Proteins via Enhanced Non-Covalent Bonding. Front. Cell Dev. Biol..

[67] Hopfinger M.C., Kirkpatrick C.C., Znosko B.M. (2020). Predictions and Analyses of RNA Nearest Neighbor Parameters for Modified Nucleotides. Nucleic Acids Res..

[68] Shi Y., Lei Y., Chen M., Ma H., Shen T., Zhang Y., Huang X., Ling W., Liu S.-Y., Pan Y. (2024). A Demethylation-Switchable Aptamer Design Enables Lag-Free Monitoring of m^6^ A Demethylase FTO with Energy Self-Sufficient and Structurally Integrated Features. J. Am. Chem. Soc..

[69] Murray M.T., Wetmore S.D. (2024). Unlocking Precision in Aptamer Engineering: A Case Study of the Thrombin Binding Aptamer Illustrates Why Modification Size, Quantity, and Position Matter. Nucleic Acids Res..

[70] Saito Y., Hudson R.H.E. (2018). Base-Modified Fluorescent Purine Nucleosides and Nucleotides for Use in Oligonucleotide Probes. J. Photochem. Photobiol. C Photochem. Rev..

[71] Sun Y., Zhang N., Han C., Chen Z., Zhai X., Li Z., Zheng K., Zhu J., Wang X., Zou X. (2021). Competitive Immunosensor for Sensitive and Optical Anti-Interference Detection of Imidacloprid by Surface-Enhanced Raman Scattering. Food Chem..

[72] Liu C., Li Y., Chen T., Meng S., Liu D., Dong D., You T. (2022). Electric Field-Induced Specific Preconcentration to Enhance DNA-Based Electrochemical Sensing of Hg^2+^ via the Synergy of Enrichment and Self-Cleaning. J. Agric. Food Chem..

[73] Wu D., Gordon C.K.L., Shin J.H., Eisenstein M., Soh H.T. (2022). Directed Evolution of Aptamer Discovery Technologies. Acc. Chem. Res..

[74] Minagawa H., Onodera K., Fujita H., Sakamoto T., Akitomi J., Kaneko N., Shiratori I., Kuwahara M., Horii K., Waga I. (2017). Selection, Characterization and Application of Artificial DNA Aptamer Containing Appended Bases with Sub-Nanomolar Affinity for a Salivary Biomarker. Sci. Rep..

[75] Kong L.-Z., Kim S.-M., Wang C., Lee S.Y., Oh S.-C., Lee S., Jo S., Kim T.-D. (2023). Understanding Nucleic Acid Sensing and Its Therapeutic Applications. Exp. Mol. Med..

[76] Kimoto M., Cox R.S., Hirao I. (2011). Unnatural Base Pair Systems for Sensing and Diagnostic Applications. Expert. Rev. Mol. Diagn..

[77] Chen Y., Kong D., Qiu L., Wu Y., Dai C., Luo S., Huang Z., Lin Q., Chen H., Xie S. (2022). Artificial Nucleotide Aptamer-Based Field-Effect Transistor for Ultrasensitive Detection of Hepatoma Exosomes. Anal. Chem..

[78] Leconte A.M., Hwang G.T., Matsuda S., Capek P., Hari Y., Romesberg F.E. (2008). Discovery, Characterization, and Optimization of an Unnatural Base Pair for Expansion of the Genetic Alphabet. J. Am. Chem. Soc..

[79] Kimoto M., Hirao I. (2020). Genetic Alphabet Expansion Technology by Creating Unnatural Base Pairs. Chem. Soc. Rev..

[80] Matsunaga K., Kimoto M., Lim V.W., Tan H.P., Wong Y.Q., Sun W., Vasoo S., Leo Y.S., Hirao I. (2021). High-Affinity Five/Six-Letter DNA Aptamers with Superior Specificity Enabling the Detection of Dengue NS1 Protein Variants beyond the Serotype Identification. Nucleic Acids Res..

[81] Futami K., Kimoto M., Lim Y.W.S., Hirao I. (2019). Genetic Alphabet Expansion Provides Versatile Specificities and Activities of Unnatural-Base DNA Aptamers Targeting Cancer Cells. Mol. Ther. Nucleic Acids.

[82] Miao S., Liang Y., Rundell S., Bhunia D., Devari S., Munyaradzi O., Bong D. (2021). Unnatural Bases for Recognition of Noncoding Nucleic Acid Interfaces. Biopolymers.

[83] Boldyrev A.I., Wang L.-S. (2005). All-Metal Aromaticity and Antiaromaticity. Chem. Rev..

[84] Banti C.N., Tsiatouras V., Karanicolas K., Panagiotou N., Tasiopoulos A.J., Kourkoumelis N., Hadjikakou S.K. (2020). Antiproliferative Activity and Apoptosis Induction, of Organo-Antimony(III)–Copper(I) Conjugates, against Human Breast Cancer Cells. Mol. Divers..

[85] Harlepp S., Chardon E., Bouché M., Dahm G., Maaloum M., Bellemin-Laponnaz S. (2019). N-Heterocyclic Carbene-Platinum Complexes Featuring an Anthracenyl Moiety: Anti-Cancer Activity and DNA Interaction. Int. J. Mol. Sci..

[86] Zhao Q., Han B., Peng C., Zhang N., Huang W., He G., Li J. (2024). A Promising Future of Metal-*N*-heterocyclic Carbene Complexes in Medicinal Chemistry: The Emerging Bioorganometallic Antitumor Agents. Med. Res. Rev..

[87] Chen D., Hua Y., Xia H. (2020). Metallaaromatic Chemistry: History and Development. Chem. Rev..

[88] Wang Z., Liu Z., Zhang W., Li Y., Feng Y., Lv S., Diao H., Luo Z., Yan P., He M. (2023). AptaDiff: De Novo Design and Optimization of Aptamers Based on Diffusion Models. Brief. Bioinform..

[89] Dauparas J., Lee G.R., Pecoraro R., An L., Anishchenko I., Glasscock C., Baker D. (2025). Atomic Context-Conditioned Protein Sequence Design Using LigandMPNN. Nat. Methods.

[90] Bashir A., Yang Q., Wang J., Hoyer S., Chou W., McLean C., Davis G., Gong Q., Armstrong Z., Jang J. (2021). Machine Learning Guided Aptamer Refinement and Discovery. Nat. Commun..

[91] Mousivand M., Javan-Nikkhah M., Anfossi L., Di Nardo F., Salina M., Bagherzadeh K. (2023). High Performance Aptasensing Platform Development through in Silico Aptamer Engineering for Aflatoxin B1 Monitoring. Food Control.

[92] Mo Y., Wu K., Zheng Y., Ma N., Wang Z., Wu S., Duan N. (2025). In Silico-Guided Engineering of Broad-Spectrum Aptamer for the Highly Sensitive Detection of Organophosphate Pesticides. Chem. Eng. J..

[93] Wong F., He D., Krishnan A., Hong L., Wang A.Z., Wang J., Hu Z., Omori S., Li A., Rao J. (2024). Deep Generative Design of RNA Aptamers Using Structural Predictions. Nat. Comput. Sci..

[94] Zacco E., Kantelberg O., Milanetti E., Armaos A., Panei F.P., Gregory J., Jeacock K., Clarke D.J., Chandran S., Ruocco G. (2022). Probing TDP-43 Condensation Using an in Silico Designed Aptamer. Nat. Commun..

[95] Moussa S., Kilgour M., Jans C., Hernandez-Garcia A., Cuperlovic-Culf M., Bengio Y., Simine L. (2023). Diversifying Design of Nucleic Acid Aptamers Using Unsupervised Machine Learning. J. Phys. Chem. B.

[96] Driscoll J., Gondaliya P., Ziemer A., Yan I.K., Gupta Y., Patel T. (2024). In Silico Design of Novel EpCAM-Binding Aptamers for Targeted Delivery of RNA Therapeutics. Nanomaterials.

[97] Yang Y., Qiao L., Jiang Y., Wang Z., Zhang D., Buratto D., Huang L., Zhou R. (2025). In Silico Design and Validation of High-Affinity RNA Aptamers for SARS-CoV-2 Comparable to Neutralizing Antibodies. bioRxiv.

[98] Sun D., Sun M., Zhang J., Lin X., Zhang Y., Lin F., Zhang P., Yang C., Song J. (2022). Computational Tools for Aptamer Identification and Optimization. TrAC Trends Anal. Chem..

[99] Hamada M. (2018). In Silico Approaches to RNA Aptamer Design. Biochimie.

[100] Liu Y., Le C., Tyrrell D.L., Le X.C., Li X.-F. (2020). Aptamer Binding Assay for the E Antigen of Hepatitis B Using Modified Aptamers with G-Quadruplex Structures. Anal. Chem..

[101] Stangherlin S., Ding Y., Liu J. (2024). Dissociation Constant ( *K*_d_ ) Measurement for Small-Molecule Binding Aptamers: Homogeneous Assay Methods and Critical Evaluations. Small Methods.

[102] Sullivan R., Adams M.C., Naik R.R., Milam V.T. (2019). Analyzing Secondary Structure Patterns in DNA Aptamers Identified via CompELS. Molecules.

[103] Gong T., Ju F., Bu D. (2024). Accurate Prediction of RNA Secondary Structure Including Pseudoknots through Solving Minimum-Cost Flow with Learned Potentials. Commun. Biol..

[104] Gao H., Ding Y., Ping P., Wang D., Ma Y., Li H. (2024). Signal-on Electrogenerated Chemiluminescence Detection of Gonyautoxin 1/4 Based on Proximity Ligation-Induced an Electrode-Bound Pseudoknot DNA. Talanta.

[105] de-los-Santos-Álvarez N., Miranda-Ordieres A.J., Tuñón-Blanco P. (2008). Aptamers as Recognition Elements for Label-Free Analytical Devices. TrAC Trends Anal. Chem..

[106] de-los-Santos-Álvarez N., Lobo-Castañón M.J., Miranda-Ordieres A.J., Tuñón-Blanco P. (2008). TrAC Trends in Analytical Chemistry.

[107] Zhao Q., Yu X., Zhou C., Yagoub A.E.A., Ma H. (2020). Effects of Collagen and Casein with Phenolic Compounds Interactions on Protein in Vitro Digestion and Antioxidation. LWT.

[108] Zhao D., Dong X., Jiang N., Zhang D., Liu C. (2014). Selective Recognition of Parallel and Anti-Parallel Thrombin-Binding Aptamer G-Quadruplexes by Different Fluorescent Dyes. Nucleic Acids Res..

[109] Jing M., Bowser M.T. (2011). Methods for Measuring Aptamer-Protein Equilibria: A Review. Anal. Chim. Acta.

[110] Troisi R., Napolitano V., Rossitto E., Osman W., Nagano M., Wakui K., Popowicz G.M., Yoshimoto K., Sica F. (2023). Steric Hindrance and Structural Flexibility Shape the Functional Properties of a Guanine-Rich Oligonucleotide. Nucleic Acids Res..

[111] Wang Z., Zhang F., Fang L., Chen F., Yang W., Wang Z. (2024). Development of Folding-Based and Signal-Amplified Fluorescence Aptasensors Using G-Quadruplex Quinclorac Aptamer Variants Engineered via Exonuclease Digestion and Circular Dichroism Spectroscopy. Sens. Actuators B Chem..

[112] Wallace B.A. (2009). Protein Characterisation by Synchrotron Radiation Circular Dichroism Spectroscopy. Quart. Rev. Biophys..

[113] Nam Y., Cho D., Gu B., Rouxel J.R., Keefer D., Govind N., Mukamel S. (2022). Time-Evolving Chirality Loss in Molecular Photodissociation Monitored by X-Ray Circular Dichroism Spectroscopy. J. Am. Chem. Soc..

[114] Pilicer S.L., Dragna J.M., Garland A., Welch C.J., Anslyn E.V., Wolf C. (2020). High-Throughput Determination of Enantiopurity by Microplate Circular Dichroism. J. Org. Chem..

[115] Wang C., Zhu K., Shi P., Ding X., Zhang S. (2022). Rapid and Label-Free Detection of Aflatoxin B1 Using a Rationally Truncated Aptamer and via Circular Dichroism Measurement. Chem. Commun..

[116] Vinegrad E., Vestler D., Ben-Moshe A., Barnea A.R., Markovich G., Cheshnovsky O. (2018). Circular Dichroism of Single Particles. ACS Photonics.

[117] Tai T., Sha F., Wang X., Wang X., Ma K., Kirlikovali K.O., Su S., Islamoglu T., Kato S., Farha O.K. (2022). Leveraging Isothermal Titration Calorimetry to Explore Structure–Property Relationships of Protein Immobilization in Metal–Organic Frameworks. Angew. Chem. Int. Ed..

[118] Li W., Sun S., Chen W., Ma H., Li T., Zhang Z., Wu D., Yan M., Yang Y. (2024). Exploring the Taste Presentation and Receptor Perception Mechanism of Salty Peptides from *Stropharia Rugosoannulata* Based on Molecular Dynamics and Thermodynamics Simulation. Food Sci. Hum. Wellness.

[119] Yu H., Zhao Q. (2024). Sensitive Electrochemical Sensor for Cd^2+^ with Engineered Short High-Affinity Aptamer Undergoing Large Conformation Change. Talanta.

[120] Zhang S., Ning Z., Zhang Y., Lin X., Duan N., Wang Z., Wu S. (2025). Construction of a Microfluidic SELEX Platform for Efficient Screening of Advanced Glycation End Products Aptamer. Biosens. Bioelectron..

[121] Zhang Y., Xu Z., Xiao Y., Jiang H., Zuo X., Li X., Fang X. (2024). Structural Mechanisms for Binding and Activation of a Contact-Quenched Fluorophore by RhoBAST. Nat. Commun..

[122] Jia W., Guo A., Zhang R., Shi L. (2023). Mechanism of Natural Antioxidants Regulating Advanced Glycosylation End Products of Maillard Reaction. Food Chem..

[123] Karonen M. (2025). Polyphenol–Macromolecule Interactions by Isothermal Titration Calorimetry. Macromol..

[124] Adam A.A., Michaux F., Dos Santos Morais R., Seiler A., Muniglia L., Khanji A.N., Jasniewski J. (2024). Determination of the Critical Aggregation Concentration in Water of Gum Arabic Functionalized with Curcumin Oxidation Products by Micro-Scale Thermophoresis Approach. Int. J. Biol. Macromol..

[125] Li F., Zhang Z., Shi Q., Wang R., Ji M., Chen X., Li Y., Liu Y., Yu S. (2025). Thermal Proteome Profiling (TPP) Reveals NAMPT as the Anti-Glioma Target of Phenanthroindolizidine Alkaloid PF403. Acta Pharm. Sin. B.

[126] Yu H., Zhao Q. (2023). Sensitive Microscale Thermophoresis Assay for Rapid Ochratoxin A Detection with Fluorescently Labeled Engineered Aptamer. Analyst.

[127] Basu S., Gohain N., Kim J., Trinh H.V., Choe M., Joyce M.G., Rao M. (2023). Determination of Binding Affinity of Antibodies to HIV-1 Recombinant Envelope Glycoproteins, Pseudoviruses, Infectious Molecular Clones, and Cell-Expressed Trimeric Gp160 Using Microscale Thermophoresis. Cells.

[128] Jerabek-Willemsen M., André T., Wanner R., Roth H.M., Duhr S., Baaske P., Breitsprecher D. (2014). MicroScale Thermophoresis: Interaction Analysis and Beyond. Journal of Molecular Structure.

[129] Liu J., Ren Z., Sun Y., Xu L., Wei D., Tan W., Ding D. (2023). Investigation of the Relationship between Aptamers’ Targeting Functions and Human Plasma Proteins. ACS Nano.

[130] Yu H., Zhao Q. (2023). Profiling Additional Effects of Aptamer Fluorophore Modification on Microscale Thermophoresis Characterization of Aptamer–Target Binding. Anal. Chem..

[131] Yu H., Zhao Q. (2024). Microscale Thermophoresis Sensor for Cd2+ Using Aptamer with Tetramethylrhodamine Label at an Internal Site. Sensors and Actuators B: Chemical.

[132] Yu H., Zhao Q. (2022). Sensitive Microscale Thermophoresis Assay Using Aptamer Thermal Switch. Anal. Chem..

[133] Yu H., Zhao Q. (2023). DNAzyme-Based Microscale Thermophoresis Sensor. Anal. Chem..

[134] Garoli D., Yamazaki H., Maccaferri N., Wanunu M. (2019). Plasmonic Nanopores for Single-Molecule Detection and Manipulation: Toward Sequencing Applications. Nano Lett..

[135] Greiss F., Kriegel F., Braun D. (2017). Probing the Cooperativity of Binding Networks with High-Throughput Thermophoresis. Anal. Chem..

[136] Zhao Q., Tao J., Uppal J.S., Peng H., Wang H., Le X.C. (2019). Nucleic Acid Aptamers Improving Fluorescence Anisotropy and Fluorescence Polarization Assays for Small Molecules. TrAC Trends Anal. Chem..

[137] Zhao Q., Tao J., Feng W., Uppal J.S., Peng H., Le X.C. (2020). Aptamer Binding Assays and Molecular Interaction Studies Using Fluorescence Anisotropy—A Review. Anal. Chim. Acta.

[138] Wang L., Haruna S.A., Ahmad W., Wu J., Chen Q., Ouyang Q. (2022). Tunable Multiplexed Fluorescence Biosensing Platform for Simultaneous and Selective Detection of Paraquat and Carbendazim Pesticides. Food Chem..

[139] Wang Y., Li W., Hu X., Zhang X., Huang X., Li Z., Li M., Zou X., Shi J. (2021). Efficient Preparation of Dual-Emission Ratiometric Fluorescence Sensor System Based on Aptamer-Composite and Detection of Bis(2-Ethylhexyl) Phthalate in Pork. Food Chem..

[140] Selva Sharma A., Marimuthu M., Varghese A.W., Wu J., Xu J., Xiaofeng L., Devaraj S., Lan Y., Li H., Chen Q. (2024). A Review of Biomolecules Conjugated Lanthanide Up-Conversion Nanoparticles-Based Fluorescence Probes in Food Safety and Quality Monitoring Applications. Crit. Rev. Food Sci. Nutr..

[141] Megalathan A., Wijesinghe K.M., Dhakal S. (2021). Single-Molecule FRET-Based Dynamic DNA Sensor. ACS Sens..

[142] Zhang C., Yu X., Shi X., Han Y., Guo Z., Liu Y. (2020). Development of Carbon Quantum Dot–Labeled Antibody Fluorescence Immunoassays for the Detection of Morphine in Hot Pot Soup Base. Food Anal. Methods.

[143] Li D., Chen Z., Mei X. (2017). Fluorescence Enhancement for Noble Metal Nanoclusters. Adv. Colloid. Interface Sci..

[144] Wang C., Gu C., Zhao X., Yu S., Zhang X., Xu F., Ding L., Huang X., Qian J. (2024). Self-Designed Portable Dual-Mode Fluorescence Device with Custom Python-Based Analysis Software for Rapid Detection via Dual-Color FRET Aptasensor with IoT Capabilities. Food Chem..

[145] Li H., Huang X., Mehedi Hassan M., Zuo M., Wu X., Chen Y., Chen Q. (2020). Dual-Channel Biosensor for Hg^2+^ Sensing in Food Using Au@Ag/Graphene-Upconversion Nanohybrids as Metal-Enhanced Fluorescence and SERS Indicators. Microchem. J..

[146] Zhang J., Wang L., Jäschke A., Sunbul M. (2021). A Color-Shifting Near-Infrared Fluorescent Aptamer–Fluorophore Module for Live-Cell RNA Imaging. Angew. Chem. Int. Ed..

[147] Gaillard T. (2018). Evaluation of AutoDock and AutoDock Vina on the CASF-2013 Benchmark. J. Chem. Inf. Model..

[148] Vu H., Gilari K., Sathiyamoorthy V., Beckham J. (2022). Discovery of Novel Inhibitors for *Mycobacterium tuberculosis* D-alanine: D-alanine Ligase through Virtual Screening. FASEB J..

[149] Ropii B., Bethasari M., Anshori I., Koesoema A.P., Shalannanda W., Satriawan A., Setianingsih C., Akbar M.R., Aditama R., Fahmi F. (2024). The Molecular Interaction of Six Single-Stranded DNA Aptamers to Cardiac Troponin I Revealed by Docking and Molecular Dynamics Simulation. PLoS ONE.

[150] Ye H., Wang M., Yu X., Ma P., Zhu P., Zhong J., He K., Guo Y. (2023). Molecular Docking Insight into the Label-Free Fluorescence Aptasensor for Ochratoxin A Detection. Molecules.

[151] Zhang C., Wang L., Tu Z., Sun X., He Q., Lei Z., Xu C., Liu Y., Zhang X., Yang J. (2014). Organophosphorus Pesticides Detection Using Broad-Specific Single-Stranded DNA Based Fluorescence Polarization Aptamer Assay. Biosens. Bioelectron..

[152] Shmilovich K., Chen B., Karaletsos T., Sultan M.M. (2023). DEL-Dock: Molecular Docking-Enabled Modeling of DNA-Encoded Libraries. J. Chem. Inf. Model..

[153] Ivanova A., Mokshyna O., Polishchuk P. (2024). StreaMD: The Toolkit for High-Throughput Molecular Dynamics Simulations. J. Cheminform.

[154] Fedulova A.S., Armeev G.A., Romanova T.A., Singh-Palchevskaia L., Kosarim N.A., Motorin N.A., Komarova G.A., Shaytan A.K. (2024). Molecular Dynamics Simulations of Nucleosomes Are Coming of Age. WIREs Comput. Mol. Sci..

[155] Lappala A. (2024). The next Revolution in Computational Simulations: Harnessing AI and Quantum Computing in Molecular Dynamics. Curr. Opin. Struct. Biol..

[156] Wang P., Li H., Hassan M.M., Guo Z., Zhang Z.-Z., Chen Q. (2019). Fabricating an Acetylcholinesterase Modulated UCNPs-Cu^2+^ Fluorescence Biosensor for Ultrasensitive Detection of Organophosphorus Pesticides-Diazinon in Food. J. Agric. Food Chem..

[157] Yang Y., Tang Y., Wang C., Liu B., Wu Y. (2021). Selection and Identification of a DNA Aptamer for Ultrasensitive and Selective Detection of λ-Cyhalothrin Residue in Food. Anal. Chim. Acta.

[158] Li W., Hu X., Li Q., Shi Y., Zhai X., Xu Y., Li Z., Huang X., Wang X., Shi J. (2020). Copper Nanoclusters @ Nitrogen-Doped Carbon Quantum Dots-Based Ratiometric Fluorescence Probe for Lead (II) Ions Detection in Porphyra. Food Chem..

[159] Khoshbin Z., Housaindokht M.R., Izadyar M., Bozorgmehr M.R., Verdian A. (2019). Theoretical Design and Experimental Study of New Aptamers with the Improved Target-Affinity: New Insights into the Pb^2+^-Specific Aptamers as a Case Study. J. Mol. Liq..

[160] Qian S.Q., Yuan M., Zuo X.W., Cao H., Yu J.S., Hao L.-L., Yang K.L., Xu F. (2024). A Novel Strategy for Enhancing the Stability of Aptamer Conformations in Heavy Metal Ion Detection. Anal. Chim. Acta.

[161] Komijani M., Shamabadi N.S., Shahin K., Eghbalpour F., Tahsili M.R., Bahram M. (2021). Heavy Metal Pollution Promotes Antibiotic Resistance Potential in the Aquatic Environment. Environ. Pollut..

[162] Liu Z., Wang X., Ren X., Li W., Sun J., Wang X., Huang Y., Guo Y., Zeng H. (2021). Novel Fluorescence Immunoassay for the Detection of Zearalenone Using HRP-Mediated Fluorescence Quenching of Gold-Silver Bimetallic Nanoclusters. Food Chem..

[163] Guan Y., Ma J., Neng J., Yang B., Wang Y., Xing F. (2023). A Novel and Label-Free Chemiluminescence Detection of Zearalenone Based on a Truncated Aptamer Conjugated with a G-Quadruplex DNAzyme. Biosensors.

[164] Wang J., Zhu L., Li T., Li C., Zhang W., Sun X., Yue X., Xu W. (2024). In Silico Simulation-Guided Engineering of Multifunctional Bivalent Light up Aptamer for Sensitive and on-Site Detection of AFB1 Using Label-Free Ratiometric Fluorescent Aptasensor. Sens. Actuators B Chem..

[165] Gao Y.-N., Wu C.-Q., Wang J.-Q., Zheng N. (2022). Metabolomic Analysis Reveals the Mechanisms of Hepatotoxicity Induced by Aflatoxin M1 and Ochratoxin A. Toxins.

[166] Jin J., Kouznetsova V.L., Kesari S., Tsigelny I.F. (2023). Synergism in Actions of HBV with Aflatoxin in Cancer Development. Toxicology.

[167] Pourakbari R., Shadjou N., Yousefi H., Isildak I., Yousefi M., Rashidi M.-R., Khalilzadeh B. (2019). Recent Progress in Nanomaterial-Based Electrochemical Biosensors for Pathogenic Bacteria. Microchim. Acta.

[168] Yang N., Ding N., Qi S., Shang Z., Ma P., Khan I.M., Wang Z., Xia Y., Zhang Y., Zhang L. (2024). High-Affinity Truncated Aptamers for Detection of *Cronobacter* Spp with Magnetic Separation-Assisted DNAzyme-Driven 3D DNA Walker. Microchim. Acta.

[169] Sun M., Ma N., Shi H., Cheong L.-Z., Yang W., Qiao Z. (2023). A HCR Based Multivalent Aptamer Amplifier for Ultrasensitive Detection of Salmonella. Sens. Actuators B Chem..

[170] Tang Y., Li Y., Chen P., Zhong S., Yang Y. (2025). Nucleic Acid Aptamer-Based Sensors for Bacteria Detection: A Review. BioEssays.

[171] Yu Y., Zhang L., Qin Z., Karges J., Xiao H., Su X. (2023). Unraveling and Overcoming Platinum Drug-Resistant Cancer Tumors with DNA Nanostructures. Adv. Funct. Mater..

[172] Amero P., Lokesh G.L.R., Chaudhari R.R., Cardenas-Zuniga R., Schubert T., Attia Y.M., Montalvo-Gonzalez E., Elsayed A.M., Ivan C., Wang Z. (2021). Conversion of RNA Aptamer into Modified DNA Aptamers Provides for Prolonged Stability and Enhanced Antitumor Activity. J. Am. Chem. Soc..

[173] Chen X., Duan M., Chang Y., Ye M., Wang Z., Wu S., Duan N. (2024). Assembly of a Multivalent Aptamer for Efficient Inhibition of Thermostable Direct Hemolysin Toxicity Induced by Vibrio Parahaemolyticus. J. Hazard. Mater..

[174] Han E., Pan Y., Li L., Cai J. (2023). Bisphenol A Detection Based on Nano Gold-Doped Molecular Imprinting Electrochemical Sensor with Enhanced Sensitivity. Food Chem..

[175] Jia M., Sha J., Li Z., Wang W., Zhang H. (2020). High Affinity Truncated Aptamers for Ultra-Sensitive Colorimetric Detection of Bisphenol A with Label-Free Aptasensor. Food Chem..

[176] Wu S., Liu S., Wang Z., Chen Y., Zhao G. (2023). Comprehensive Analysis of Bisphenol Analogues in Complex Water Using a Group-Targeting Aptamer Engineered by Base Mutation. J. Hazard. Mater..

[177] Ben Aissa S., Mastouri M., Catanante G., Raouafi N., Marty J.L. (2020). Investigation of a Truncated Aptamer for Ofloxacin Detection Using a Rapid FRET-Based Apta-Assay. Antibiotics.

[178] Guo H., Sun Y., Ma P., Khan I.M., Duan N., Wang Z. (2022). Sensitive Detection of Patulin Based on DNase Ⅰ-Assisted Fluorescent Aptasensor by Using AuNCs-Modified Truncated Aptamer. Food Control.

[179] Sun A., Qi S., Li Y., Wu Y., Zhang Y., Wang Z. (2025). Ultra-Stable MAPbBr3@ZIF-8-Based Fluorescent Aptasensor for Highly Sensitive and Specific Detection of Azlocillin in Food. Food Biosci..

[180] Ma P., Guo H., Ye H., Zhang Y., Wang Z. (2023). Aptamer-Locker Probe Coupling with Truncated Aptamer for High-Efficiency Fluorescence Polarization Detection of Zearalenone. Sens. Actuators B Chem..

[181] Ma P., Guo H., Duan N., Ma X., Yue L., Gu Q., Wang Z. (2021). Label Free Structure-Switching Fluorescence Polarization Detection of Chloramphenicol with Truncated Aptamer. Talanta.

[182] Zhang W., Sun Z., Tian Y., Mou Y., Guo Y., Sun X., Li F. (2024). Ratiometric Fluorescent Sensor Based on a Truncated Specific Aptamer by MGO-SELEX Screening for Streptomycin Detection. Sens. Actuators B Chem..

[183] Chen X., Yang L., Tang J., Wen X., Zheng X., Chen L., Li J., Xie Y., Le T. (2022). An AuNPs-Based Fluorescent Sensor with Truncated Aptamer for Detection of Sulfaquinoxaline in Water. Biosensors.

[184] He A., Wan L., Zhang Y., Yan Z., Guo P., Han D., Tan W. (2024). Structure-Based Investigation of a DNA Aptamer Targeting PTK7 Reveals an Intricate 3D Fold Guiding Functional Optimization. Proc. Natl. Acad. Sci. USA.

[185] Han J., Ma P., Khan I.M., Zhang Y., Wang Z. (2023). Study of Binding Mechanism of Aptamer to Kanamycin and the Development of Fluorescent Aptasensor in Milk Detection. Talanta.

[186] Rahman M.S., Han M.J., Kim S.W., Kang S.M., Kim B.R., Kim H., Lee C.J., Noh J.E., Kim H., Lee J.-O. (2023). Structure-Guided Development of Bivalent Aptamers Blocking SARS-CoV-2 Infection. Molecules.

[187] Gawande B.N., Rohloff J.C., Carter J.D., Von Carlowitz I., Zhang C., Schneider D.J., Janjic N. (2017). Selection of DNA Aptamers with Two Modified Bases. Proc. Natl. Acad. Sci. USA.

[188] Pfeiffer F., Rosenthal M., Siegl J., Ewers J., Mayer G. (2017). Customised Nucleic Acid Libraries for Enhanced Aptamer Selection and Performance. Curr. Opin. Biotechnol..

[189] Kähkölä H., Herath M., Virta P., Lönnberg T. (2025). Post-SELEX Modification of Quinine Aptamers through Neoacetalization. Org. Biomol. Chem..

